# Comprehensive Nutritional and Dietary Intervention for Autism Spectrum Disorder—A Randomized, Controlled 12-Month Trial

**DOI:** 10.3390/nu10030369

**Published:** 2018-03-17

**Authors:** James B. Adams, Tapan Audhya, Elizabeth Geis, Eva Gehn, Valeria Fimbres, Elena L. Pollard, Jessica Mitchell, Julie Ingram, Robert Hellmers, Dana Laake, Julie S. Matthews, Kefeng Li, Jane C. Naviaux, Robert K. Naviaux, Rebecca L. Adams, Devon M. Coleman, David W. Quig

**Affiliations:** 1 Arizona State University, School for Engineering of Matter, Transport & Energy, Tempe, AZ 85287, USA; autismstudynurseasu@gmail.com (E.G.); ecgehn@gmail.com (E.G.); Valeria.Fimbres@asu.edu (V.F.); epollard1025@gmail.com (E.L.P.); julieaingram@yahoo.com (J.I.); thebeckyadams@gmail.com (R.L.A.); devon.coleman@asu.edu (D.M.C.); 2 Health Diagnostics, South Amboy, NJ 08879, USA; audhyatk@optonline.net; 3 Southwest College of Naturopathic Medicine, Tempe, AZ 85282, USA; J.Mitchell@scnm.edu; 4 Arizona Allergy Associates, Phoenix, AZ 85004, USA; rhellmers@aol.com; 5 Dana Laake Nutrition, Kensington, MD 20895, USA; danalaake@aol.com; 6 Nourishing Hope, San Francisco, CA 94117, USA; julie@NourishingHope.com; 7 University of California, The Mitochondrial and Metabolic Disease Center, San Diego, CA 92093, USA; kli@ucsd.edu (K.L.); jnaviaux@ucsd.edu (J.C.N.); naviaux@ucsd.edu (R.K.N.); 8 Doctor’s Data, St. Charles, IL 60174, USA; dquig@DoctorsData.com

**Keywords:** autism, autism spectrum disorder, vitamins, minerals, essential fatty acids, carnitine, Epsom salts, digestive enzymes

## Abstract

This study involved a randomized, controlled, single-blind 12-month treatment study of a comprehensive nutritional and dietary intervention. Participants were 67 children and adults with autism spectrum disorder (ASD) ages 3–58 years from Arizona and 50 non-sibling neurotypical controls of similar age and gender. Treatment began with a special vitamin/mineral supplement, and additional treatments were added sequentially, including essential fatty acids, Epsom salt baths, carnitine, digestive enzymes, and a healthy gluten-free, casein-free, soy-free (HGCSF) diet. There was a significant improvement in nonverbal intellectual ability in the treatment group compared to the non-treatment group (+6.7 ± 11 IQ points vs. −0.6 ± 11 IQ points, *p* = 0.009) based on a blinded clinical assessment. Based on semi-blinded assessment, the treatment group, compared to the non-treatment group, had significantly greater improvement in autism symptoms and developmental age. The treatment group had significantly greater increases in EPA, DHA, carnitine, and vitamins A, B2, B5, B6, B12, folic acid, and Coenzyme Q10. The positive results of this study suggest that a comprehensive nutritional and dietary intervention is effective at improving nutritional status, non-verbal IQ, autism symptoms, and other symptoms in most individuals with ASD. Parents reported that the vitamin/mineral supplements, essential fatty acids, and HGCSF diet were the most beneficial.

## 1. Introduction

Many studies have demonstrated that children and adults with ASD often have significant nutritional deficiencies, metabolic imbalances, and digestive problems. Several nutritional and dietary treatment studies have demonstrated benefits in treating these underlying conditions [1,2,3]. In the following sections we discuss specific research related to vitamins, minerals, essential fatty acids, mitochondrial disorders/carnitine issues, and gastrointestinal disorders (digestive problems and food sensitivities).

### 1.1. Vitamins/Minerals

Several studies suggest that customized vitamin/mineral supplementation is beneficial for children with ASD. Three studies have demonstrated that children with ASD have impaired methylation, decreased glutathione, and increased oxidative stress [4,5,6]. Those studies demonstrated that nutritional supplementation (with methyl-B12, folinic acid, and trimethylglycine) is beneficial. Several other studies have also demonstrated increased oxidative stress [7,8,9,10,11,12]. Methylation is important because that controls epigenetics, and there is evidence that there are many differentially methylated regions in the brains of children with ASD vs. controls [13].

In 2008/2009 we conducted an extensive comparison of the nutritional and metabolic status of children with ASD (*n* = 55) compared to neurotypical children of similar age and gender (*n* = 44) [14]. Study measurements included vitamins, biomarkers of vitamin status, minerals, plasma amino acids, plasma glutathione, neurotransmitters, and biomarkers of oxidative stress, methylation, sulfation and energy production. Many statistically significant differences (*p* < 0.001) were observed in the ASD group compared to the neurotypical group, including: low levels of biotin, glutathione, methylation status (*S*-adenosylmethionine (SAM) and uridine), ATP, NADH, NADPH, sulfate (free and total), tryptophan, and GABA; also, high levels of oxidative stress markers and plasma glutamate. 

That study was followed by a three-month randomized, double-blind, placebo-controlled treatment study involving a customized vitamin/mineral supplement [15]. The supplement was found to be well-absorbed and result in many significant improvements in metabolic status, including SAM, reduced glutathione, ratio of oxidized glutathione to reduced glutathione (GSSG:GSH), nitrotyrosine, ATP, NADH, and NADPH. Most of these metabolic biomarkers improved to normal or near-normal levels. However, although free and total plasma sulfate levels improved, they remained below normal, suggesting that additional treatments are needed to fully normalize sulfation. That study also found that the supplement group had significantly greater improvements than the placebo group on autism-related symptoms on the Parental Global Impressions-Revised Average Change (*p* = 0.008), and on the subscores for Hyperactivity (*p* = 0.003), Tantrumming (*p* = 0.009), Overall (*p* = 0.02), and Receptive Language (*p* = 0.03). 

### 1.2. Essential Fatty Acids

Several polyunsaturated fatty acids (PUFAs) are either essential or conditionally essential, including several omega-3 and omega-6 fatty acids. Meta-analyses of many studies have demonstrated that omega-3 levels are decreased in certain psychiatric disorders including schizophrenia (meta-analysis of 14 studies) [16], ADHD (9 studies) [17]), depression (14 studies) [18], bipolar disorder (six studies) [19], and dementia (10 studies) [20].

Meta-analyses of many clinical trials have demonstrated benefits of supplementation with omega-3 PUFA’s for schizophrenia (10 trials) [21], ADHD (16 trials) [17], major depression (12 trials) [22], bipolar depression (5 trials) [23], and possibly dementia (eight of 13 trials positive) [24]. Meta-analyses findings indicated that EPA was more beneficial than DHA, and that higher levels of EPA were more beneficial. There is also one study [25] that found omega-3 fatty acid supplementation is very helpful for infants with Rett’s syndrome, a disorder which often includes autistic symptoms.

PUFA’s may also play a role in some gastrointestinal problems, since they are important for intestinal membrane function. One epidemiological study found that increasing incidence of Crohn’s disease correlated very strongly (*r* = 0.79) with low levels of omega-3 fatty acids [26]. A one-year, double-blind, placebo-controlled trial of fish oil (2.7 g/day of omega-3 fatty acids) in people with Crohn’s disease found that subjects taking the fish oil had a significantly reduced relapse rate, with no significant adverse effects [27]. Gastrointestinal problems are common in ASD [14,28], and PUFA supplementation may be beneficial for reducing some gastrointestinal problems in children with ASD.

A meta-analysis [29] of fifteen case-control studies (*n* = 1193) found that, compared with typically developed individuals, the ASD group had lower eicosapentaenoic acid (EPA), docosahexaenoic acid (DHA) and arachidonic acid (AA), and a lower ratio of total omega-3 to total omega-6 fatty acids; these differences were primarily found in studies with children, and not in studies with adolescents or adults. A meta-analysis [29] of four small randomized controlled trials (*n* = 107) [30,31,32,33] found that compared with placebo, omega-3 fatty acid supplementation improved social withdrawal (*p* < 0.02) and restricted interests and behaviors (*p* = 0.05), but did not have a significant effect on communication, irritability, or hyperactivity (all rated per the Aberrant Behavior Checklist). These studies only lasted 6–16 weeks, so were too short to observe full effect, since omega-3 supplementation requires about six months to reach steady-state levels in erythrocytes, and about 1–1.5 months for half of that change to occur [34]. These studies used doses of 0.5–1.5 g/day of omega-3 fatty acids. Two other small randomized studies [35,36] not included in the meta-analysis [29] did not find significant effects on symptoms despite longer duration (6 months), possibly due to small sizes (under 35 participants completed each study) or low dose (200 mg DHA) in one study [36]. 

Overall, it appears that omega-3 fatty acids are decreased in ASD, and that supplementation may be helpful. Higher doses and longer treatment may result in greater benefit. For example, a treatment study for Crohn’s disease found that long-term treatment (12 months) may be needed for improvement in gastrointestinal problems. We hypothesize that children with ASD who do not regularly eat seafood (the major source of omega-3 fatty acids in most western diets) are more likely to benefit from fish oil supplementation.

### 1.3. Sulfate

Sulfur is the fourth most common mineral in the body [37]. Most sulfate is produced in vivo by metabolism of cysteine [14]. Sulfation is important for many reactions including detoxification, inactivation of catecholamines, synthesis of brain tissue, sulfation of mucin proteins which line the gastrointestinal tract, and more. Low free and total plasma sulfate in children with ASD has been previously reported in three studies [14,38,39], and is consistent with four studies [38,40,41,42] which found that children with ASD, compared to controls, had a significantly decreased sulfation capacity, based on decreased ability to detoxify paracetamol (acetaminophen). The finding of low plasma sulfate is also consistent with a large study that found high sulfate in the urine of children with ASD [43], as sulfate wasting in the urine partly explains low levels in the plasma. ATP is required for the kidneys to resorb sulfate, and one study [14] found that plasma ATP was low in children with ASD and moderately correlated with levels of free and total plasma sulfate (*r* = 0.32 and 0.44, respectively), suggesting, that low levels of ATP are a contributor to decreased sulfate in children with ASD. One study [43], also reported high levels of urinary sulfite in children with ASD, suggesting that there was a problem of converting sulfite to sulfate in the mitochondria. In 38% of cases (14/38) urinary sulfite and sulfate levels improved by giving 50 mcg of molybdenum, based upon molybdenum dependence of the enzyme necessary for converting sulfite to sulfate (sulfite oxidase). Another study [15] found that a vitamin/mineral supplement (containing molybdenum) was able to improve, but not normalize, free and total plasma sulfate in children with ASD. Overall, these studies suggest that sulfate is low in children with ASD, and that vitamin/mineral supplementation is helpful but additional sources of sulfate (such as Epsom salt baths) are needed.

### 1.4. Carnitine and Mitochondrial Disorders

Carnitine is a conditionally essential nutrient that is vital in energy production and fatty acid metabolism. Carnitine carries long-chain fatty acids (fuel) into the mitochondria, and it also carries potentially toxic organic acids out of the mitochondria and cell so they can be eliminated from the body. Several studies have suggested that mitochondrial disorders are common in children with ASD [44,45,46,47,48]. Note that the term “mitochondrial disorders” is used to denote a generalized impairment of mitochondrial function, and are generally not as severe as “mitochondrial diseases”, which involve specific severe genetic abnormalities. One study found decreased levels of carnitine in children with ASD [49]. A recent double-blind, placebo-controlled 3-month study (*n* = 30) found that supplementation with carnitine was beneficial [50]. Specifically, the study found significantly greater improvements in the Childhood Autism Rating Scale 2 (CARS-2) and Clinical Global Impressions (CGI) scores in the treatment group compared to the placebo group. In addition, scores significantly improved in cognition and marginally in speech on the Autism Treatment Evaluation Checklist (ATEC). l-carnitine therapy significantly increased serum carnitine concentrations, and significant correlations between changes in serum free-carnitine levels and positive clinical changes were observed. l-carnitine therapy was generally well-tolerated by study subjects. A second study [51] found similar results. 

Overall, the literature suggests that mitochondrial disorders are common in ASD, and that a combination of therapy with vitamins, minerals, CoEnzyme Q10, essential fatty acids, and l-carnitine may be helpful in improving mitochondrial function.

### 1.5. Gastrointestinal Problems, Digestive Enzymes, Limited Diets, and Food Sensitivities

Gastrointestinal problems are common in children with ASD, especially chronic constipation, diarrhea, abdominal pain, and gastrointestinal inflammation [28,52]. A study by our group [53] found that those problems are strongly correlated to autism severity (*r* = 0.59, *p* < 0.001), suggesting that it is important to investigate them. As reported, in those with ASD, gastrointestinal problems appear to be partly due to deficiencies in digestive enzymes, partly due to food sensitivities, and possibly (as discussed above) due to low levels of omega-3 fatty acids, which in turn, could result in abnormal gut bacteria [54]. One large study by Horvath et al. evaluated disaccharidase activity from endoscopic biopsies in 90 children with ASD. They found that 49% had at least one deficient enzyme activity, and 20% had deficiencies in two or more disaccharidase enzymes “Lactase and maltase deficiencies were the most frequent, followed by low activity of sucrase, palatinase, and glucoamylase. All of the children with low enzyme activity had loose stools and/or gaseousness”. Another large study [55] involving intestinal biopsy samples of 199 children and adults with ASD (ages 22 months to 28 years) found that many had deficiencies in disaccharidases (enzymes for digesting simple sugars). Specifically, they found that 62% had deficiencies in lactase, 16% were deficient in sucrase, and 10% were deficient in maltase. The problems seemed to be equally common in children and adults, suggesting that these problems are lifelong. An open-label treatment study of 46 children and adults with ASD reported a wide range of benefits from the use of digestive enzymes [56], but results of randomized controlled trials are mixed [57,58]. Some studies have suggested that children with ASD have poor diets, leading to decreased intake of key nutrients [59,60].

Several studies have found that children with ASD have abnormal immune responses to certain foods, especially glutens (in wheat, rye, barley, oats) and casein (in dairy products) and sometimes soy. One study [61] found that many children with ASD have food sensitivities. Four studies [62,63,64,65] found that children with ASD had more hypersensitivities to food allergens than did typical children, and may be related to increased intestinal permeability [65,66]. A large study of 150 children with ASD found that 87% had IgG antibodies (sensitivity) to gluten, vs. 1% of the age and gender-matched controls, and 90% had IgG antibodies to casein, vs. 7% of the controls [67].

Several studies suggest that special diets can be beneficial for individuals with ASD. One open-label study [68] found that an 8-week diet which avoided allergic foods resulted in benefits in an open study of 36 children with ASD. One long-term open-label study of 70 children with ASD who followed a gluten-free, casein-free diet for one year or longer found that 81% improved significantly by the third month, with improvements continuing over the next 12 months. Large improvements were observed in social isolation, eye contact, mutism, learning skills, hyperactivity, stereotypic activity, and panic attacks [67]. A single-blind study of 10 children with autism found that 8 benefitted from a gluten-free, casein-free (GFCF) diet [69]. A 12-week, double-blind, cross-over study of a GFCF diet in 15 children with ASD did not find significant benefits, but parents reported benefits that were not identified by the testing [70]. However, a 12-month, randomized, single-blind, placebo-controlled GFCF diet study involving 54 children with ASD found statistically significant benefits in communication subscores (Autism Diagnostic Observation Schedule (ADOS) evaluation) in the GFCF diet group compared to the control group [71]. The parents (who were not blinded) also reported benefits in social interaction, daily living skills, inattention, and hyperactivity.

Overall, these studies suggest that children with ASD often have deficiencies in lactase and other digestive enzymes, may have poor diets, often have food sensitivities, especially to gluten and casein, and hence may benefit from digestive enzymes, healthier diets, and/or gluten-free, casein-free diets [65,66].

### 1.6. Study Goal

The goal of this study is to investigate a comprehensive nutritional and dietary intervention to treat children and adults with ASD. Each of these treatments have been previously studied individually and found to have some benefit, mostly in short-term studies. The goal of this study is to investigate the effect of the combination of those treatments in a long-term study. Combinations of these treatments are commonly used for treating children and adults with ASD, their effects are expected to be synergistic, and longer-term treatment may lead to greater benefits. An unusually wide age range was used in this study because this study was funded primarily by families participating in our annual walk fundraiser, and they have requested treatment studies that address all ages. This study is not designed to look at the effect of individual treatments (most of them have already been investigated individually); rather the goal is to investigate the effect of a combination of treatments which provide comprehensive, synergistic nutritional support.

### 1.7. Hypothesis

A combination of nutritional and dietary interventions will be effective in reducing the symptoms of autism, reducing gastrointestinal problems, and increasing overall functioning level.

## 2. Methods

### 2.1. Study Design and Justification

This was a one-year, single-blinded study, involving a treatment group and a non-treated group of children and adults with ASD. Single-blind means that the clinical evaluators were blinded, but the participants were not. The reason for this design is that we wanted to conduct a long-term study on the effects of a comprehensive set of dietary and nutritional treatments, similar to what the Autism Research Institute has recommended for years [72]. We believe that length of time is too long for a double-blind, placebo-controlled format, because too many participants would drop out. Also, it is extremely difficult to do a double-blind study when making major diet changes, especially the switch to a “healthy” diet as described below. A single-blind study is still a rigorous study design for assessments for which the clinical observer is fully blinded, and the additional data from unblinded participants provides some useful information similar to an open-label study. Since this was an exploratory new treatment, the choice of sample size was roughly estimated based on previous studies. The authors confirm that all ongoing and related trials for this drug/intervention are registered (Clinicialtrials.gov: NCT02059577).

### 2.2. Participant Enrollment

The study was advertised by email to approximately 2500 ASD families in Arizona, using the contact list of the Autism Society of Greater Phoenix and the Autism/Asperger’s Research Program at Arizona State University (ASU). Interested ASD families attended a one-hour informational meeting, and consenting families joined the study. Neurotypical families were recruited from friends of the ASD families and professionals who work with ASD families. Participants were recruited for the study from October 2011 to April 2014. All subjects gave their informed consent for inclusion before they participated in the study. The study was conducted in accordance with the Declaration of Helsinki, and the protocol was approved by the Institutional Review Board (IRB) of Arizona State University.

### 2.3. Enrollment Criteria—ASD Group

1. Diagnosis of autism spectrum disorder (autism, Pervasive Developmental Disorder-Not Otherwise Specified (PDD-NOS), or Asperger’s) by a psychiatrist, psychologist, or developmental pediatrician.
2. Verification of diagnosis by ASU staff based on the ADOS and/or CARS-2.
3. Age of 2.5–60 years.
4. No major changes in behavioral or medical treatments in the previous two months, and no intention to make such changes during the 12 months of the study.
5. No usage of nutritional supplements (vitamins, minerals essential fatty acids, carnitine) or special diets in the previous two months.

### 2.4. Enrollment Criteria—Neurotypical Group

1. No diagnosed mental disorders, including autism spectrum disorders, Attention Deficit Hyperactivity Disorder (ADHD), depression, anxiety, etc.
2. No first-degree relatives of individuals with ASD (no siblings or parents).
3. Age of 2.5–60 years.
4. No usage of nutritional supplements (vitamins, minerals, essential fatty acids, carnitine) or special diets in the previous two months.

Note that there was no exclusion for individuals with ASD with specific metabolic or genetic disorders. No participants reported unusual genetic or metabolic disorders, but there was no attempt to screen for those, and it is likely that some may have existed. 

### 2.5. Participants

The characteristics of the study participants are listed in Table 1. The ASD treatment group, ASD non-treatment group, and the neurotypical controls have similar age distributions (mostly children, some teens, and a few adults), and similar gender distributions (mostly male). The ASD treatment and non-treatment group have similar diagnoses (mostly autism). For the ASD group, 100% met the criteria for ASD per the CARS-2, and 88% met the criteria for ASD per the ADOS (most of the 8 participants who met only the CARS-2 criteria were high-functioning teens/adults who in the clinical judgement of the evaluator were clearly on the ASD spectrum, so they were admitted to the study).

### 2.6. Randomization

After enrollment and ADOS/CARS-2/Reynolds Intellectual Assessment Scales (RIAS)/Severity of Autism Scale (SAS-Pro) assessment, participants were randomly assigned to either the Treatment or Non-treatment group. The study coordinator enrolled participants, conducted the randomization, and assigned participants to interventions. She was not involved in any of the evaluations. The Treatment group began treatment immediately, whereas the Non-treatment group was asked not to make any changes in medical, nutritional, therapy, or education treatment for 12 months. The Non-treatment group was promised that they would receive all the supplements and diet advice at the end of the study if they made no major changes to any educational interventions for 12 months, which helped minimize the drop-out rate. Participants were enrolled on a rolling basis, and participants with similar ages were matched and then randomly assigned to one of the two groups.

### 2.7. Protocols

#### 2.7.1. Protocol for ASD Treatment Group

Initial evaluation of autism severity and overall functioning level.

Physical examination by the study physician to verify that the participant is in sufficient good health to participate in the study. Initial blood draw and first-morning urine collection.

Day 0: Vitamin/Mineral supplementation begins.

Day 30: Essential Fatty Acid supplementation begins.

Day 60: Epsom salt baths begin.

Day 90: Carnitine Supplementation begins.

Day 180: Digestive Enzyme supplementation begins.

Day 210: Healthy, casein-free, gluten-free diet begins.

Day 365: Final assessment of autism severity and overall functioning status. Final blood draw and urine collection.

#### 2.7.2. Protocol for ASD Non-Treatment Group

Initial evaluation of autism severity and overall functioning level.

Physical examination by study physician to verify that participant is in sufficient good health to participate in the study. Initial blood draw and first-morning urine collection. 

Day 365: Verification of no changes in treatment during last 12 months. Final assessment of autism severity and overall functioning status. Final blood draw and urine collection. 

#### 2.7.3. Protocol for Neurotypical Group

Physical examination by study physician to verify that participant is in sufficient good health to participate in the study. Initial blood draw and first-morning urine collection. 

### 2.8. Biomarker Measurements

Biomarkers in blood and urine were measured at the beginning and end of the study in the children and adults in the ASD groups, and one time at the start of the study in the neurotypical group. The samples were sent in a blinded fashion to the laboratories for testing.

Some testing was done by LabCorp, and some by Doctor’s Data. Both commercial laboratories are approved by the Clinical Laboratory Improvement Amendments (CLIA) program operated by the US Department of Health and Human Services which oversees approximately 200,000 laboratories in the US. Samples were taken by courier from our medical office to the local LabCorp testing facility in Phoenix (refrigerated or on dry ice as appropriate). Samples for Doctor’s Data were shipped overnight, either with cold packs or on dry ice as appropriate. 

LabCorp conducted standard measurements of blood chemistry, Complete Blood Count (CBC) with differential, ammonia, lactic acid, creatine kinase, and a thyroid panel (TSH, T3, T4).

Doctor’s Data conducted measurements of Red Blood Cell (RBC) elements and urinary iodine using the same methods as reported in a previous study [14]. Doctor’s Data also measured RBC fatty acids, C-Reactive Protein, and homocysteine-related metabolites (homocysteine, cysteine, methionine). 

RBC fatty acids were measured by gas chromatography using a flame ionization detector. Red Blood Cells were washed and derivatized to their methyl esters and extracted and separated according to carbon number.

- High sensitivity C-Reactive Protein was measured using an immunoturbidimetric method using Kamiya Reagents and analyzed on a Beckman Coulter AU680 Chemistry Analyzer (Brea, CA, USA)
- Homocysteine was measured by LC/MS after reduction using dithiothreitol and derivatization.

Vitamins and carnitine were measured at UC-San Diego by some of our team using liquid chromatography tandem mass spectrometry (LC-MS/MS) (SCIEX, Redwood City, CA, USA) as previously described with modifications [73] (PMID 25705365). The absolute concentrations of acylcarnitines were calculated using stable isotope internal standards. The levels of vitamin and co-factors were normalized using neurotypical control baseline values and reported as peak area ratios.

### 2.9. Handgrip Strength

Handgrip strength, an indicator of muscle strength, was assessed by using a pneumatic, adjustable squeeze pinch-gauge/dynamometer (Baseline Evaluation Instruments; White Plains, NY, USA) by a study nurse unaware of the treatment status of the subject. This instrument is a reliable and valid method for obtaining muscle force measurements in children and adults, and takes only a few min. One of three bulb sizes was used, depending on the participant’s hand size, and the same size was used at beginning and end of the study. Each participant was shown how to squeeze the bulb with one hand, and then three measurements were taken, and the highest value was recorded.

### 2.10. Autism Severity and Overall Functioning Assessments

A large number of assessments of autism severity and overall functioning were used because we hypothesized that there might be improvements in many different areas. Most assessments were conducted for the autism treatment and non-treatment groups at the beginning and end of the study. The PGI-2 was assessed at month, 3, 6, 9, and 12 for the treatment group, and at month 12 for the non-treatment group. The initial assessments were conducted before randomization, so neither the participants nor the evaluator knew which group they were in. At the end of the study, the evaluator first conducted the ADOS and RIAS assessments in a blinded manner. The evaluator then conducted the CARS-2 and SAS-Pro assessments, which did involve some discussion with the parents (except for a few high-functioning participants who did not have a parent available). The Vineland was conducted by phone by a different blinded evaluator. The blinding of the evaluators at the beginning and end of the study was complete in all cases except for one CARS-2/SAS-Pro assessment at the end of the study (a participant inadvertently commented about the treatment).

Autism Diagnostic Observation Schedule (ADOS): The ADOS is a 1-h structured interaction and is one of the primary tools used for clinical diagnosis of autism and autism spectrum disorders. It involved a blinded evaluation by clinicians certified in ADOS assessment.

Reynolds Intellectual Assessment Scales (RIAS): The RIAS assesses verbal and non-verbal IQ and memory. It involves a 20–30 min blinded assessment by clinicians using a variety of standardized IQ and memory activities.

Childhood Autism Rating Scale 2 (CARS-2): The CARS-2 evaluation was conducted after the ADOS and RIAS evaluations, and was based partly on the participant’s performance and interactions on those assessments, and partly on specific interactions and interview questions with the participant and their parents. The clinician was blinded as to the participant’s treatment status, but the participants and the parents were not, so this is classified as “semi-blinded”.

Severity of Autism Scale (SAS-Pro): The SAS-Pro is a single number on a scale of 0–10 to evaluate overall severity of autism symptoms. It was evaluated by the professional evaluator after the ADOS, RIAS, and CARS-2, so it was classified as “semi-blinded”.

All of the ADOS, RIAS, CARS-2, and SAS-Pro evaluations were done by the same professional evaluator at beginning and end (either EP or JI).

Vineland Adaptive Behavior Scale II (VABS-II): The VABS-II was conducted by a phone interview with the participant’s parents (or the participants in a few cases for high-functioning adults), so it was classified as “semi-blinded”. One evaluator (RLA) conducted all the interviews at beginning and end. The calculated raw scores were then converted into an age equivalent. However, most of the questions are geared towards younger ages, and there are fewer questions for the older ages, and a difference of one point for the older participants can result in a jump of more than 1 year of developmental age. Therefore, for scoring purposes we set a maximum age for the following subscales, based on the age at which questions became sparse: Receptive—11 years; Communication—12.3 years; Written—15.3 years; Domestic—15.3 years; Play—19 years; Coping—17.8 years; Gross Motor—6.8 years; Fine Motor—6.8 years; the other subscales (Personal and Community) had a maximum of 22 years. Only 2 participants in the treatment group and 2 participants in the non-treatment group had some scores at the maximum of any subscale, except for the Gross Motor and Fine Motor subscales, which had 32% and 44% of the treatment and non-treatment group scoring at the maximum for those subscales. So, although scores for Gross Motor and Fine Motor skills are reported, they need to be interpreted cautiously.

Parents (or the participants in a few cases for high-functioning adults) completed an initial medical history form, and at the beginning and end of the study they also completed several questionnaires to assess autism and related symptoms, including the following: ATEC, Pervasive Developmental Disorders Behavior Inventory (PDD-BI), Social Responsiveness Scale (SRS), 6-item Gastrointestinal Severity Index (6-GSI), Short Sensory Profile (SSP), Aberrant Behavior Checklist (ABC), and Medical History. Also, the Parent Global Impressions-2 (PGI-2) was completed at the end of months 3, 6, 9, and 12, to assess changes during the previous 3 months.

The PGI-2 is introduced here as an expanded version of the PGI-R [14]. The PGI-2 evaluates changes in 17 areas, and overall, using a 7-point scale ranging from “much worse” to “much better”. An “Average Change” is computed by computing the average in all 18 scores of the PGI-2, but exempting areas where initial symptom severity was “none”. The PGI-2 is similar to the CGI, but conducted by a parent instead of a clinician, and specific to autism. As discussed in a previous paper [15] our experience indicates it is more reliable to ask parents directly about observed changes than to have them estimate symptom severity at beginning and end and then compute a difference. Also, the use of a 7-point scale to detect changes seems to yield a high sensitivity to changes. Note that for each symptom we only report changes if the participant had the symptom at the start of the study.

### 2.11. Treatments

#### 2.11.1. Vitamin/Mineral Supplement

This study involved an improved version of the vitamin/mineral supplement which was found to be beneficial for children and adults with ASD in a previous study [15]. The supplement from that study was slightly modified based on pre and post measurements of levels of vitamins, minerals, and other biomarkers [15]. The major changes for this study included: increases of some nutrients (vitamin D, niacin, pantothenic acid, biotin, selenium, mixed tocopherols) and decreases of others (manganese, molybdenum, lithium). Also, several new nutrients were added, including Vitamin K, potassium, carnitine, vanadium, and boron.

Table 2 lists the supplement used in this study, at a dosage for a 60-pound (27 kg) child. The dosage was adjusted up or down based on bodyweight, to a maximum of 120 pounds (54 kg). The dosage was slowly increased over 4 weeks to the level listed in Table 2. In most cases the dosage was split into three doses (breakfast, lunch, dinner), but in a few cases the families preferred to split it into two doses for convenience (breakfast and dinner).

#### 2.11.2. Essential Fatty Acids

One of the best sources of omega-3 fatty acids is fish oil, and a recent study [74] confirms the high absorption of omega-3 fatty acids from fish oil. We used a concentrated fish oil supplement, ProEFA-Xtra by Nordic Naturals, which is a blend of fish oil (for omega-3 fatty acids) and modest amounts of borage oil (for omega-6 fatty acids). Each capsule contains: 609 mg omega-3 fatty acids (425 mg EPA, 110 mg DHA, 74 mg other omega-3 fatty acids), 198 mg omega-6 fatty acids (including 128 mg GLA), and 15 mg omega-9 fatty acids. The dosage varied with body weight:

- 30–50 pounds (14–23 kg): 2 capsules/day
- 51–100 pounds (23–45 kg): 3 capsules/day
- 100+ pounds (45+ kg): 4 capsules/day

Initial dosages started at 1 capsule/day, and increased to the above dosage over 2–4 weeks.

#### 2.11.3. Epsom Salt Baths

Epsom salts are magnesium sulfate, and internal research by one of our team (TA) has found that Epsom salt baths are one of the most effective ways to raise plasma sulfate levels, which are normally low in people with ASD. Therefore, each participant was asked to take a warm bath for 20 min 2×/week, with 2 cups Epsom salt and a half cup baking soda (which increases absorption of Epsom salts) added to the bath.

#### 2.11.4. Carnitine

Each participant was given a dosage of 50 mg acetyl-l-carnitine/kg bodyweight-day, to a maximum of 2 grams/day, the same dosage as used in a previous study [50] involving l-carnitine, as that dosage was found to be beneficial and well-tolerated. The dosage was gradually increased to the full dosage over 4 weeks. Half the dosage was given in the morning, and half at dinnertime.

#### 2.11.5. Digestive Enzymes

This study involved the use of a comprehensive digestive enzyme complex for digesting food proteins (peptidase, protease 4.5, protease 3.0), carbohydrates (lactase, alpha-galactosidase, invertase, xylanase), starches (amylase, glucoamylase) and fats (lipase)—see Table 3. The dosage was one capsule for a snack or small adult meal, two capsules for a typical adult meal, and three capsules for a large adult meal. Compared to other commercial digestive enzymes, this complex is characterized as “low-medium” in protease activity, “medium” level in carbohydrase and starch-hydrolyzing activity, and “medium-high” in lipase activity.

#### 2.11.6. Healthy, Gluten-Free, Casein-Free, Soy-Free Diet

Participants were provided with written instructions about the diet, a 1-h PowerPoint presentation with audio describing the diet, a 1-h personal consult with one of our nutritionists, and the opportunity to ask additional questions from the nutritionist or study nurse. The nutritionist provided detailed advice, but the family made the final decision about meal plans and their degree of adherence to these guiding principles. The degree of compliance with each of these guiding principles was self-evaluated. 

The major guiding principles of the dietary plans included:

1. Adequate intake of a variety of vegetables (including leafy greens) and fruit (preferably whole fruit).
2. Adequate protein quality and intake.
3. Adequate, but not excessive, caloric intake.
4. Minimal consumption of “junk” foods and replacement with healthy snacks.
5. Healthy, gluten-free, casein-free, and soy-free (HGCSF).
6. Avoidance of artificial flavors, colors, and preservatives.

### 2.12. Statistical Analysis 

Different types of statistical analyses were used, depending on the research question being addressed. For comparison of changes of behavioral symptoms of the treatment vs. non-treatment group, 1-sided unpaired *t*-tests assuming unequal variance were used, since our hypothesis was that the treatment group would improve more than the non-treatment group. For comparing changes in biomarkers, 2-sided unpaired *t*-tests comparisons assuming unequal variance were used. For individual comparisons, a *p*-value of 0.05 or lower was assumed significant. No correction was made for multiple comparisons since in most cases (such as vitamin measurements) there were a large number of significant findings. This was an exploratory study, and future studies can use our results to make specific hypotheses and appropriate statistical corrections for multiple hypotheses.

### 2.13. Participant Withdrawals, Removals, and Adverse Effects

**Figure 1:**
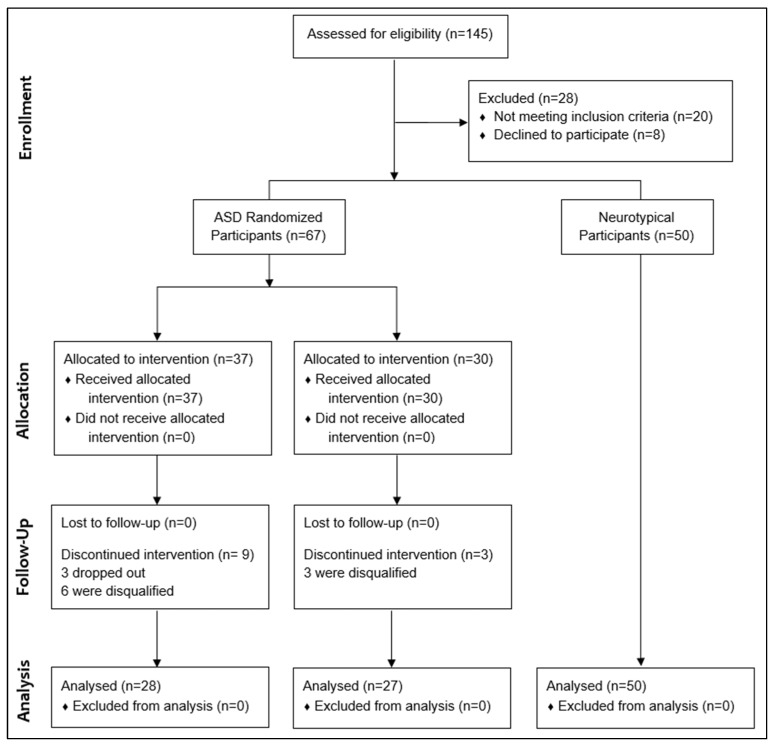
Study Flowchart.

Figure 1 displays a flow chart of the study. 67 participants with ASD began the study, and 50 neurotypical participants were assessed at baseline only.

### 2.14. Treatment Group

37 families started in the Treatment group, three dropped out, six were disqualified, and 28 completed the study

- one participant dropped after four months because of lack of benefit
- one family dropped after seven months due to insufficient benefit and disinterest in taking supplements
- one participant dropped after four months for unknown reasons
- four participants were disqualified by researchers due to poor compliance with study protocol (parents were inconsistent in giving supplements due to parental, not child, issues)
- two participants (brothers) were disqualified because they discontinued all supplements and only completed the special diet

### 2.15. Non-Treatment Group

Thirty families started in the Non-treatment group, none dropped out, three were disqualified, and 27 completed the study without making any major changes in their baseline treatments. This is an unusually high percentage for a 120 month study, primarily due to the promise of receiving a full year of free supplements if they waited.

Three participants were disqualified because they made significant changes to their baseline treatments—one made a major diet change and added three psychiatric medications; one started a developmental preschool at 10 h/week; one changed their school and home therapy program. Also, one participant did only part of the final evaluation (ADOS/CARS-2/RIAS/SAS-Pro), but no parent questionnaires or blood work due to parental issues.

## 3. Results

### 3.1. Adverse Effects

A few adverse effects were reported for some treatments.

Vitamin/Minerals: Two participants (brothers ages 7 and 12) had worsening behavior (moderate severity) that was possibly due to the vitamin/mineral supplement, so after four months they stopped use of all supplements and only implemented the healthy HGCSF diet (which was beneficial and resolved severe pica in the seven-year-old). Our measurements found that they both had extremely low cobalamin (4–5% of normal), and low levels of other nutrients compared to normal children, including methylcobalmin (40–50% of normal), beta carotene (23–36% of normal), riboflavin (46–52% of normal). The boy with pica also had low vitamin C (34% of normal), low nictotinic acid (29% of normal), and low pantothenic acid (35% of normal). The boy without pica also had low levels of folic acid (34% of normal). So, the very low level of cobalamin, and lower levels of other nutrients, may have made them very sensitive to nutritional supplements, and/or they may have an underlying metabolic problem with cobalamin. It seems likely that these nutritional deficiencies also contributed to the severe pica in one of the boys.

Carnitine: One participant reported that the carnitine made their child feel sick, so they discontinued it.

Digestive Enzymes: One participant was not able to tolerate the digestive enzyme due to intestinal symptoms, and stopped taking it after one month. One participant developed a facial rash after extended use of the digestive enzyme, and eventually discontinued it despite reporting improvements in constipation and behavior. 

Healthy HGCSF diet: One parent reported that implementation of the diet in a strict manner resulted in increased aggression towards peers, inability to problem solve, and increased spinning behavior, probably due to frustration in regards to removal of favorite foods. 

No adverse events were reported with the essential fatty acids or Epsom salt bath.

About 20% of the participants complained about the taste of the unflavored vitamin/mineral supplement and/or the fish oil, but mixing it with juice helped in most cases, and a capsule form of the vitamin/mineral supplement was made available to some families who preferred this form of delivery. A few participants had temporary mild nausea, and one child had loose stools, but lowering the dosage resolved those concerns.

### 3.2. Compliance

Compliance with taking the supplements and following the diet was self-reported by participants at the end of the study. The percentages of families who missed doses 1×/week or less was 85% for the vitamin/mineral supplement, 89% for the essential fatty acids, 82% for the carnitine, and 78% for the digestive enzymes. For the vitamin/mineral supplement, one child took only 2/3 of the full dose, and one child took ¾ of the full dose. One child did not take the carnitine supplement, and two did not take the digestive enzymes.

For compliance with a healthy diet, 7% of families reported they had only 60% compliance, 60% reported they had 80% compliance, and 33% reported they had 90% or higher compliance. Most noncompliance was reported as occurring at school or with care providers other than the primary parent. 

For compliance with a HGCSF diet, 61% reported < 1 exposure per month, 14% reported one exposure per week, 18% reported 1–2 exposures/week, and 7% reported reduced intake of gluten, casein, and soy but did not eliminate it. Families reported that compliance with the diet was the hardest part of the treatment protocol to follow.

### 3.3. Highlights

**Figure 2:**
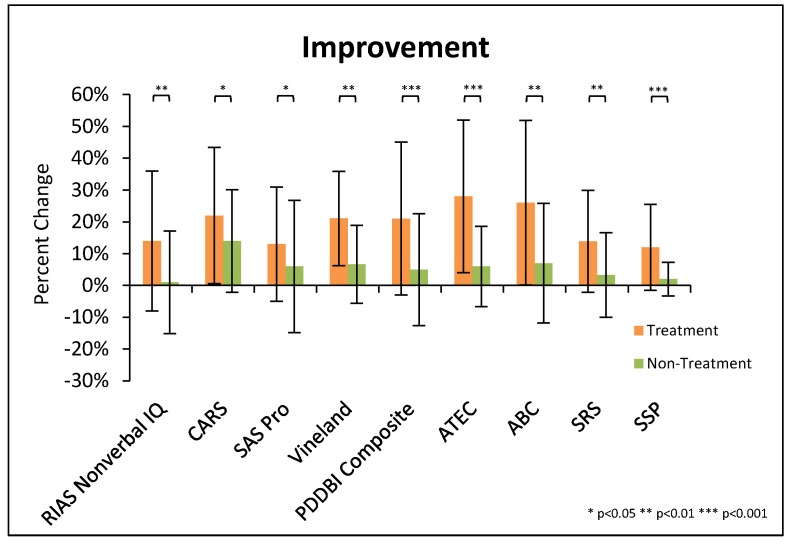
Summary of significant changes in major evaluations, for both the treatment and non-treatment groups. For some scales an increase is an improvement, and for some the opposite is true; so, here we plot them with improvement being in the same direction on the *y*-axis. Note that the % change for the PDD-BI composite is based on the average change in each of the composite subscales. Error bars represent standard deviations.

Figure 2 provides a summary of the highlights of the study. It plots the behavioral changes that were significantly different between the treatment and non-treatment groups for the major assessments.

### 3.4. Blinded Evaluations (RIAS, ADOS)

#### 3.4.1. Reynolds Intellectual Assessment Scales (RIAS) 

**Figure 3:**
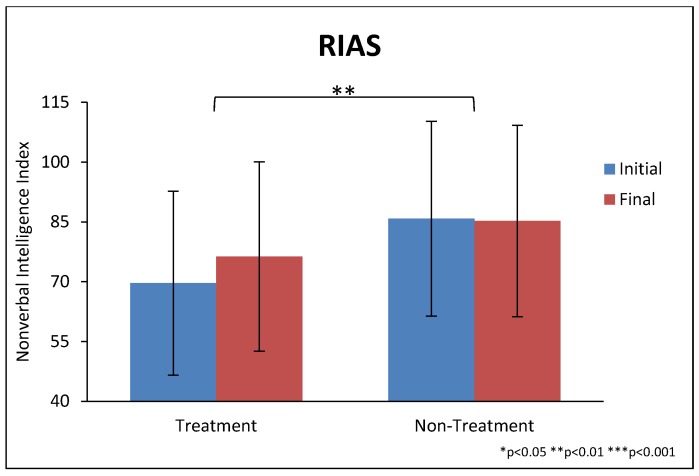
Reynolds Intellectual Assessment Scales (RIAS) nonverbal IQ score at the beginning and end of the study, for the treatment and non-treatment groups. RIAS scores are normalized so that 100 is an “average” IQ; thus, the average of the ASD groups is substantially lower than the average for the general population. Error bars represent standard deviations.

The treatment group improved significantly more than the non-treatment group on the Non-Verbal IQ test (+6.7 ± 11.4 vs. −0.6 ± 10.7, *p* = 0.009), see Table 4 and Figure 3. There was no significant difference on the Verbal IQ test or the Memory test. It is noted that at baseline the treatment group had a lower score than the non-treatment group on the Non-Verbal IQ test (*p* < 0.05), which was a random difference between the groups as a result of the randomization process. 

#### 3.4.2. Autism Diagnostic Observation Schedule (ADOS)

There was no significant change on the ADOS scores for either treatment or non-treatment group. This assessment is meant for diagnosis, and is relatively insensitive to changes since it is scored on a 3-point scale.

### 3.5. Semi-Blinded Evaluations (CARS-2, SAS-Pro, Vineland)

#### 3.5.1. Childhood Autism Rating Scale (CARS-2)

**Figure 4:**
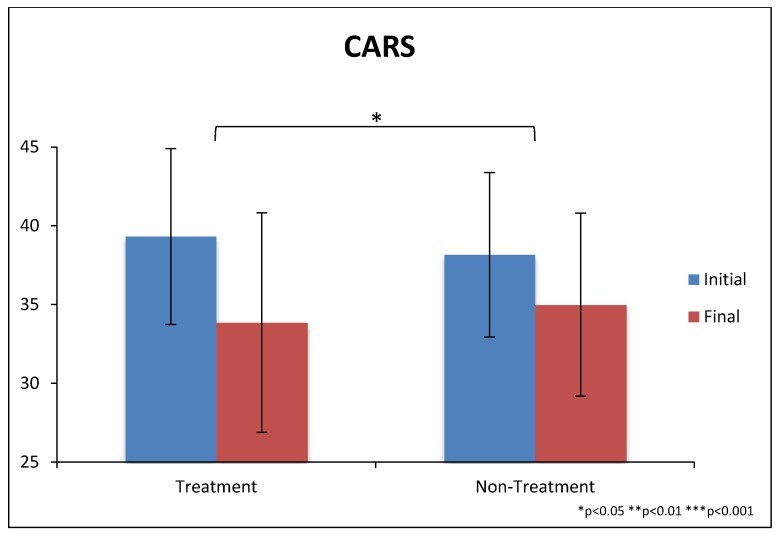
CARS-2 scores at beginning and end of the study. The scale goes from 15 to 60, with scores of approximately 27 and above being the cut-off for ASD. Error bars represent standard deviations.

The treatment group improved somewhat more than the non-treatment group on the CARS-2, and the difference was significant (−5.5 ± 5.2 vs. −3.2 ± 3.7, *p* = 0.03), see Table 4 and Figure 4. These improvements correspond to a 22% decrease vs. a 14% decrease, respectively (since the CARS-2 has a minimum score of 15, the percentages are calculated relative to the minimum score of 15).

#### 3.5.2. Severity of Autism Scale—Professional Evaluation (SAS-Pro) 

**Figure 5:**
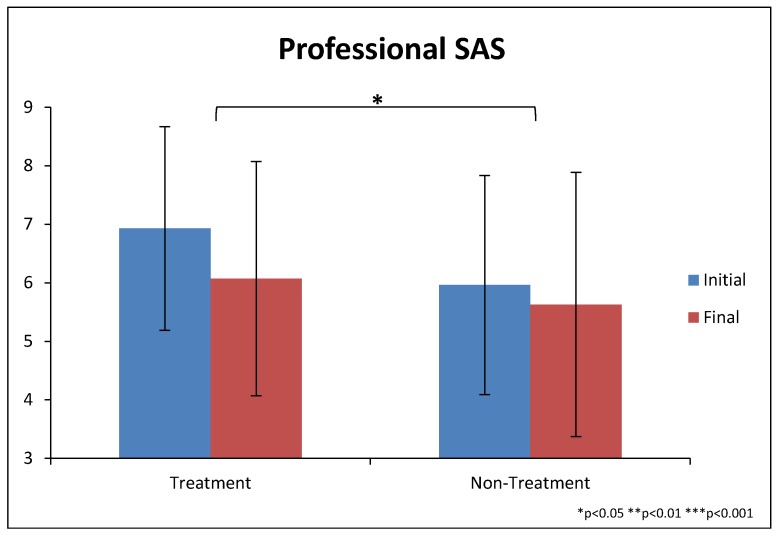
SAS scores (as rated by the professional evaluator) at beginning and end of the study. The scale goes from zero (no symptoms) to 10 (severe autism). Error bars represent standard deviations.

The treatment group improved somewhat more than the non-treatment group on the SAS-Pro as rated by our clinical evaluator, and the difference was significant (−0.93 ± 1.2 vs. −0.33 ± 0.12, *p* = 0.04), see Table 4 and Figure 5. These changes correspond to a 13% and 6% decrease in SAS-Pro scores, respectively.

The RIAS was single-blinded, and the CARS-2 and SAS-Pro were semi-blinded (evaluator was blinded, participants were not). For the RIAS, higher numbers mean more ability, with 100 being average for the general population. For the CARS-2 and SAS-Pro, higher numbers mean worse problems. #—For the CARS-2, since the lowest possible scores is a 15, the % change is relative to that baseline of 15.

#### 3.5.3. Vineland Adaptive Behavior Scale II (VABS-II) 

**Figure 6:**
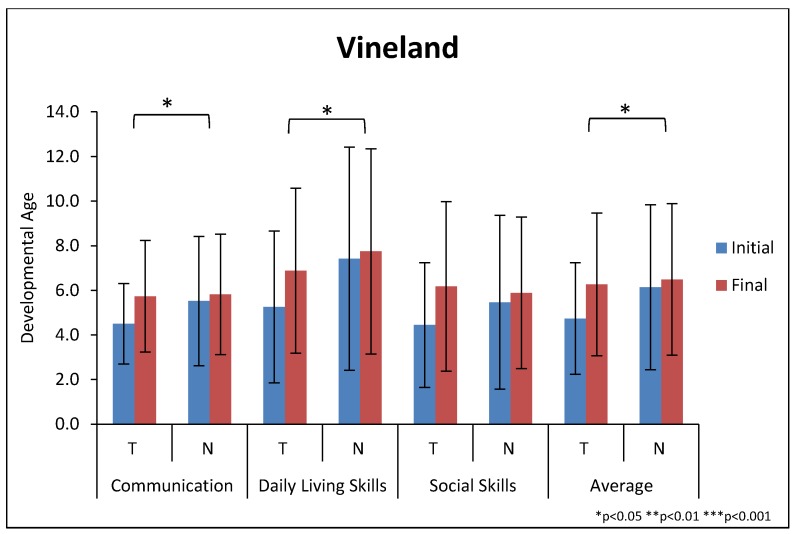
Change in the developmental age for the Vineland domains, and the average of the three domains. “T” refers to the treatment group and “N” refers to the non-treatment group. Note that the physical age of the participants at the start of the study was 10.8 and 12.3 years for the treatment and non-treatment groups, respectively. So, their developmental age was far below their physical age, even after a significant increase for the treatment group. Error bars represent standard deviations.

**Figure 7:**
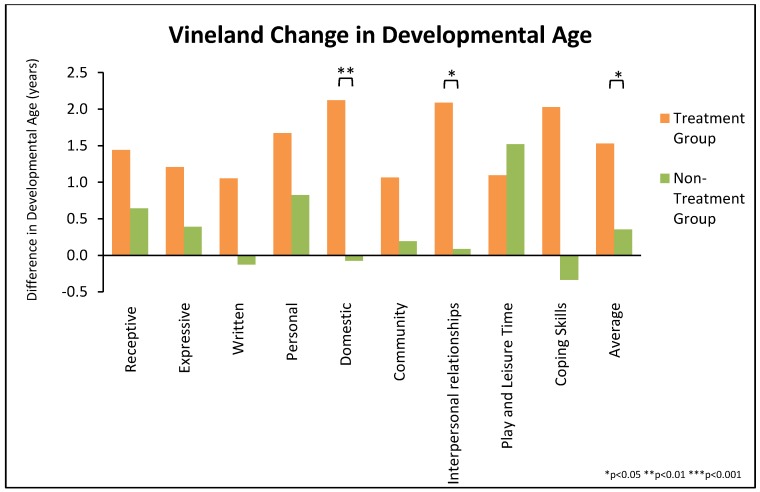
Vineland Subscale Changes.

For the average developmental age of the Communication, Social, and Daily Living domains, the treatment group improved significantly more than the non-treatment group (18.4 ± 16 months vs. 4.3 ± 16 months, *p* = 0.008), see Table 5 and Figure 6. The treatment group improved significantly more on the Communication, Daily Living Skills, and Social Skills Domains. For the 9 subscales, the treatment group improved significantly more than the non-treatment group on four of them (Written Skills, Domestic Skills, Interpersonal Relationships, Coping Skills) and marginally significant greater improvement on three others (Receptive Skills and Expressive Skills, and Community Skills), but no significant difference in Personal Daily Living Skills or Play/Leisure Skills—see Figure 7. For the Gross Motor and Fine Motor subscales both groups had similar degrees of improvement, but it is important to remember that 32% and 44% of the treatment and non-treatment groups, respectively, were at the maximum score, so they could not improve more. Due to a combination of scheduling problems and limited parental interest in the lengthy interview, pre and post VABS-II evaluations were completed on only 60% of the treatment group and 59% of the non-treatment group. However, a comparison of their PGI-2 scores shows little difference between those who did and did not complete both VABS-II evaluations, so the limited number of evaluations did not seem to bias the results.

### 3.6. Unblinded Parent/Self Evaluations

#### 3.6.1. Pervasive Developmental Disorders Behavior Inventory (PDD-BI)

**Figure 8:**
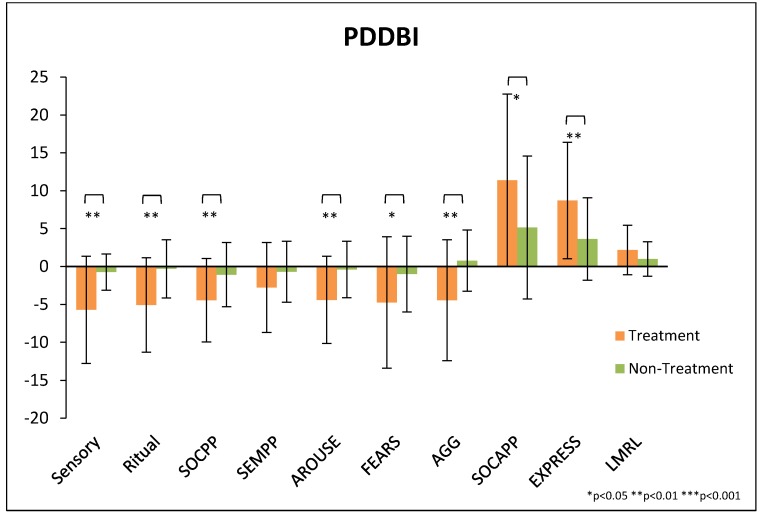
Change in PDD-BI subscale scores. Note that the first seven subscales are for maladaptive behaviors, so a decrease is beneficial. The last three subscales are for adaptive behaviors, so an increase is beneficial. Error bars represent standard deviations.

There was significantly greater improvement on the modified Autism Composite score of the PDD-BI for the treatment group compared to the non-treatment group (−35 ± 29 vs. −11 ± 17, *p* = 0.0002), see Table 6 and Figure 8. If we calculate the average of the % change on each of the subscales that compose the Autism Composite, the average % change was 21% and 5% for the treatment and non-treatment groups, respectively. The treatment group also had significantly greater improvement on most of the PDD-BI subscales.

#### 3.6.2. Autism Treatment Evaluation Checklist (ATEC)

**Figure 9:**
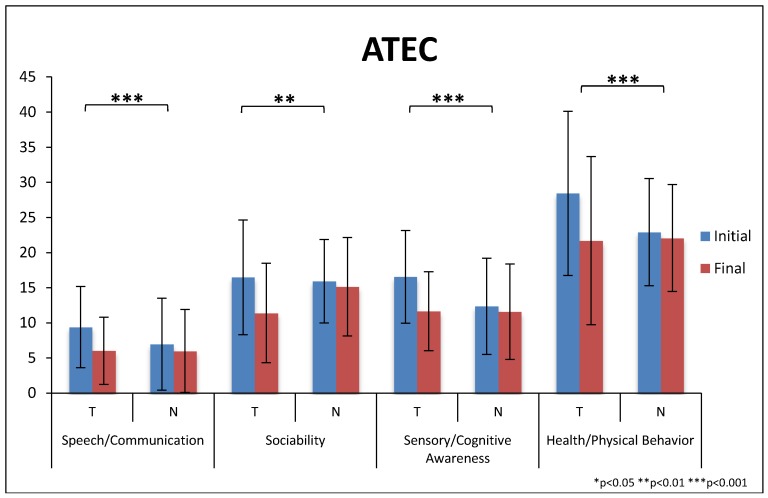
The scores for the four ATEC subscales at the beginning and end of the study. “T” refers to the treatment group and “N” refers to the non-treatment group. Higher scores represent greater severity. Error bars represent standard deviations.

There were significantly greater improvements on the total score of the ATEC for the treatment group compared to the non-treatment group (−28% vs. −6%, *p* = 0.00004), see Table 7 and Figure 9. The treatment group had significantly greater improvements on all four of the subscales.

#### 3.6.3. Aberrant Behavior Checklist (ABC)

**Figure 10:**
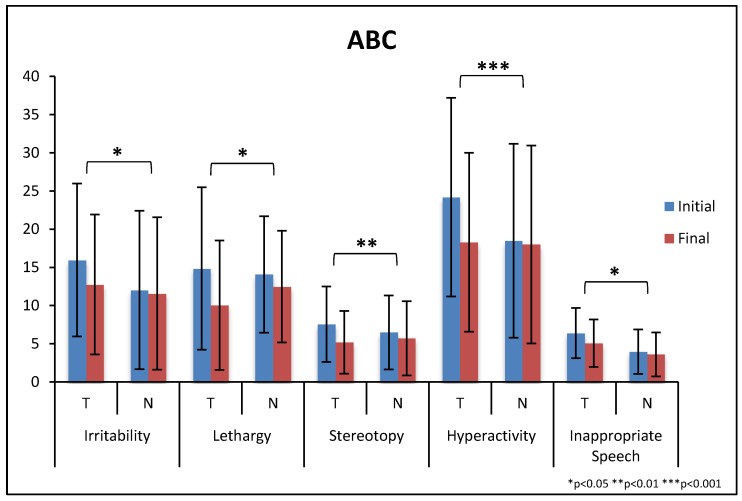
ABC subscales at beginning and end of the study. “T” refers to the treatment group and “N” refers to the non-treatment group. Higher scores represent greater severity. Error bars represent standard deviations.

There were significantly greater improvements on the total score of the ABC for the treatment group compared to the non-treatment group (−26% vs. −7%, *p* = 0.001), see Table 8 and Figure 10. The treatment group had significantly greater improvements on four of the five subscales.

#### 3.6.4. Social Responsiveness Scale (SRS)

**Figure 11:**
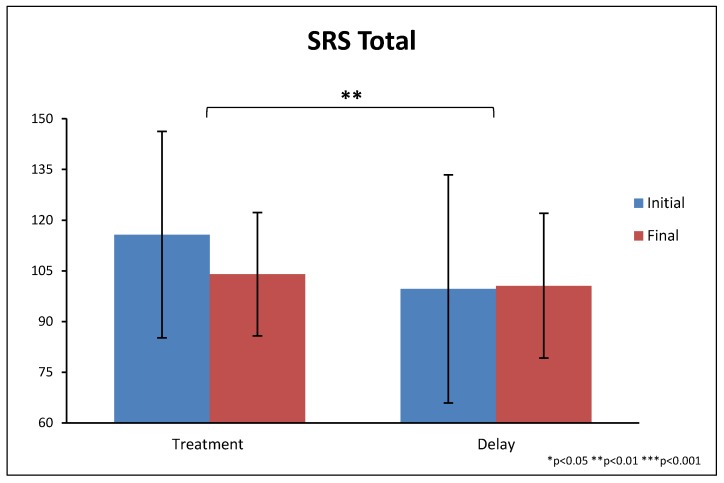
Total SRS scores at the beginning and end of the study. Higher scores indicate greater severity, and 54 is the cut-off for an ASD diagnosis. Error bars represent standard deviations.

There were significantly greater improvements on the total score of the SRS for the treatment group compared to the non-treatment group (−14% vs. −3%, *p* = 0.004), see Table 9 and Figure 11. The treatment group also had significantly greater improvements on four of the five subscales. 

#### 3.6.5. Short Sensory Profile (SSP)

**Figure 12:**
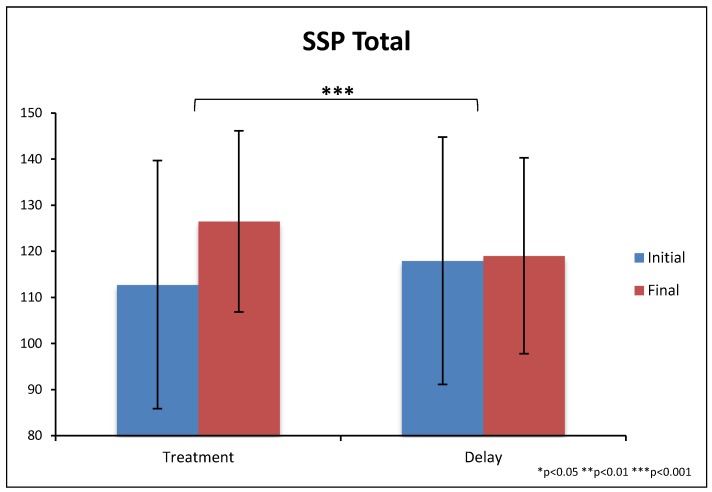
SSP scores at the beginning and end of the study. Note that higher scores represent fewer sensory problems. Error bars represent standard deviations.

There were significantly greater improvements on the total score of the SSP for the treatment group compared to the non-treatment group (12% vs. 2%, *p* = 0.0003), see Table 10 and Figure 12. The treatment group also had significantly greater improvements on five subscales (tactile sensitivity, taste/smell sensitivity, under-responsiveness/seeks sensation, auditory filtering, and visual/auditory sensitivity, *p* < 0.05), and greater but not-significant improvement on the other two subscales. 

#### 3.6.6. Parent Global Impressions—Revised-2 (PGI-2)

Table 11 lists the number of participants in each group who had each symptom. At the end of 12 months, there were significantly greater improvements on the Average Score of the PGI-2 for the treatment group compared to the non-treatment group (1.24 ± 0.74 vs. 0.08 ± 0.54, *p* < 0.00000001), see Table 11. The treatment group also had significantly greater improvements on 16 of the 17 individual areas. 

The PGI-2 assesses change in symptoms (from beginning to end of study), using a scale ranging from −3 (much worse) to zero (no change) to 1 (slightly better), 2 (better), 3 (much better). The table lists the number of participants who had the symptom at the start of the study. 

**Figure 13:**
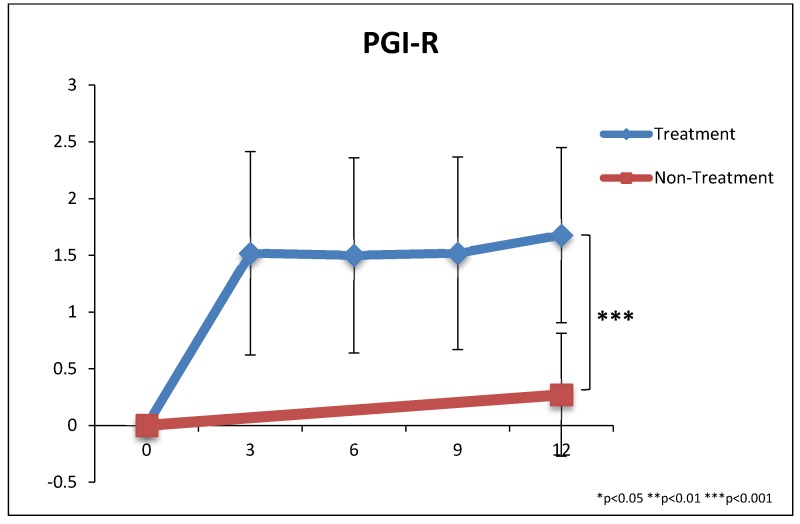
PGI-R2 scores during the study. The scale goes from −3 (much worse) to 0 (no change) to 1 (slightly better), 2 (better), 3 (much better). Error bars represent standard deviations.

PGI-2 vs. Time: The PGI-2 was also measured at 3, 6, and 9 months for the treatment group only, and the Average Score of the PGI-2 is plotted in Figure 13. Most of the improvements in symptoms occurred during the first three months, with smaller improvements after that point.

#### 3.6.7. 6-item Gastrointestinal Severity Index (6-GSI)

This analysis was limited to the participants who had non-zero Total Severity Scores on the 6-GSI at the start of the study (22 of 28 in the treatment group and 21 of 27 in the non-treatment group). The treatment group improved more than the non-treatment group on the total severity score (−30% vs. −10%, *p* = 0.05). The largest improvements were in constipation, diarrhea, and stool smell, see Table 12. Also, there were three participants who initially had zero scores on the 6-GSI, but developed some GI symptoms at the end of the study (two participants in the treatment group had a 1 and 2 point worsening, respectively, and one participant in the non-treatment group had a 4-point worsening).

### 3.7. Handgrip Strength

There was a slight increase in handgrip strength in both groups, consistent with an increase in physical development over 12 months, but no significant differences between the two groups, see Table 13.

### 3.8. Treatment Effectiveness

**Figure 14:**
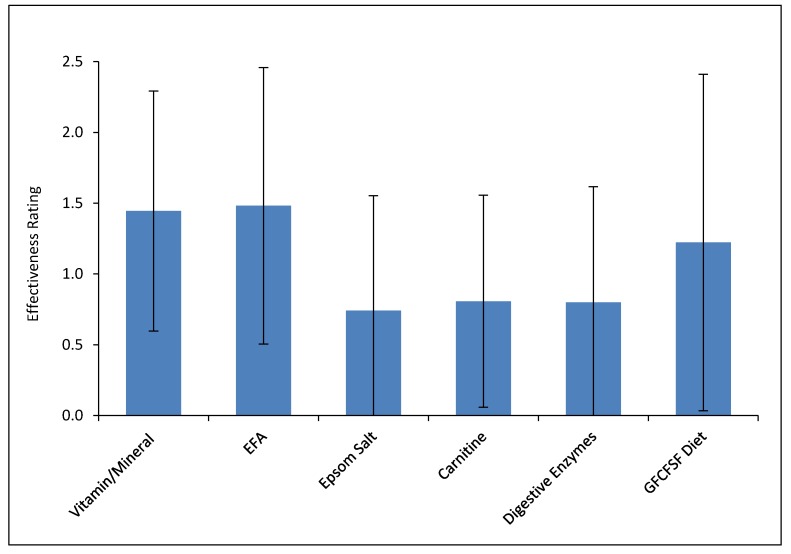
Effectiveness of each treatment as rated by parents. This is rated on a scale of −3 (much worse) to 0 (no effect) to 1 (slightly better) to 2 (better) to 3 (much better). Error bars represent standard deviations.

At the end of the study, families were asked to rate the estimated effect of each treatment, since the treatments were started at least one month apart from one another, with the caveat that some treatments may take longer than one month to have an effect so effects may overlap. Figure 14 plots the results. The highest rated treatments were the vitamin/mineral supplement and the essential fatty acids, followed by the Healthy HGCSF diets, followed by the carnitine, digestive enzymes, and Epsom salt baths. 

### 3.9. Treatment Continuation 

**Figure 15:**
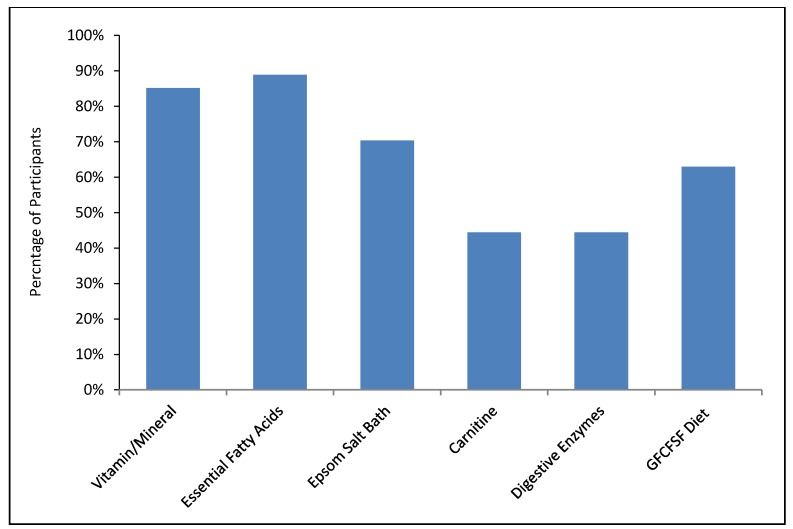
Percentage of participants who plan to continue each treatment.

At the end of the study, families were asked which treatments they were going to continue when the study ended. Figure 15 plots the data. The vitamin/mineral supplement and the essential fatty acids were the most likely to be continued (>85%). 70% of families planned to continue the Epsom salt baths, 63% planned to continue the healthy HGCSF diet, and 44% planned to continue the carnitine and digestive enzymes.

### 3.10. Medical Tests

#### 3.10.1. Complete Blood Count (CBC)

Most of the CBC measurements did not change significantly. The only significant differences were that the treatment group has a slight decrease in RBC and the non-treatment group did not change (−2% vs. 0%, *p* = 0.04), and the treatment group had a very slight increase in mean corpuscular volume (MCV) and the non-treatment group did not change (+1% vs. 0%, *p* = 0.02), see Table 14.

#### 3.10.2. Blood Chemistry Panel (ChemPanel)

Most of the ChemPanel measurements did not change significantly, see Table 15. For Blood Urea Nitrogen (BUN), the treatment group had a small decrease and the non-treatment group did not change (−16% vs. 5%, *p* = 0.01). For Serum Potassium, there was a slight decrease in the treatment group and little change in the non-treatment group (−6% vs. −1%, *p* = 0.02). 

#### 3.10.3. Body Mass Index (BMI)

There was no significant difference in the change in the BMI of the treatment group (20.7 ± 5.3 to 20.0 ± 4.6) compared to the change of the non-treatment group (20.4 ± 5.5 to 19.9 ± 5.4).

#### 3.10.4. Fatty Acids

The treatment group had large increases in eicosapentaenoic acid (EPA) and docosahexaenoic acid (DHA) compared to the non-treatment group (EPA, + 525% vs. +22%, *p* = 1 × 10^−9^, DHA: +83% vs. +13%, *p* = 1 × 10^−9^). The treatment group also had a small decrease in arachidonic acid (AA, −20% vs. −1%, *p* = 1 × 10^−7^), linoleic acid (−15% vs. −1%, *p* = 0.0001), dihomo-γ-linolenic acid (DGLA, −12% vs. +2%, *p* = 0.003), elaidic acid (−26% vs. −12%, *p* = 0.03), and palmitoleic acid (−13% vs. +11%, *p* = 0.01), see Table 16.

Low initial levels of linoleic acid were inversely correlated with parents reports of Treatment Effectiveness of the EFA supplement (*r* = −0.55, *p* < 0.001). In other words, the group reporting the most improvement on the EFA supplement tended to be those with the lowest initial levels of linoleic acid. There were no other significant correlations of improvement with the EFA supplement and levels of other PUFAs.

#### 3.10.5. C-Reactive Protein (CRP)

There was no significant difference between the change in CRP in the treatment and non-treatment groups.

#### 3.10.6. Vitamins

For the treatment group compared to the non-treatment group there were large and significant increases in biomarkers for Vitamin B2 (riboflavin, +268% vs. −17%, *p* = 0.00000002), B5 (pantothenic acid, +351% vs. +90%, *p* = 0.0002), folic acid (folic acid, +119% vs. −34%, *p* = 0.02), and CoQ10H2 (the reduced form of CoQ10, +72% vs. −19%, *p* = 0.001; no significant change in the oxidized form, CoQ10). There was a large increase in one biomarker of vitamin B6 (4-pyridoxic +435% vs. +6%, *p* = 0.0000006), but only a small increase in another biomarker (pyridoxine, +25% vs. −33%, *p* = 0.008). There were moderate increases in one form of vitamin B12 (cyanocobalamin, +44% vs. −5%, *p* = 0.006) but not in another (methylcobalamin), see Table 17. There were no significant changes in the other biomarkers.

#### 3.10.7. RBC Elements

There was a significant increase in selenium (+5% vs. −8%, *p* = 0.001) and chromium (+18% vs. −16%, *p* = 0.05) in the treatment group compared to the non-treatment group, see Table 18. There were no significant changes in other minerals.

#### 3.10.8. Homocysteine Pathway

For the treatment group compared to the non-treatment group, there was a significantly larger decrease (improvement) in homocysteine (−29% vs. −7%, *p* = 0.00002), resulting in normal levels; see Table 19. There were no significant changes in cysteine or methionine. 

#### 3.10.9. Carnitine

There was a significant increase in l-carnitine in the treatment group compared to the non-treatment group (+20% vs. −5%, *p* = 0.03), see Table 20. There was a non-significant increase in acetyl-l-carnitine in the treatment group compared to the non-treatment group (+32% vs. +4%, n.s.). The parent rating of Treatment Effectiveness for carnitine had a modest, non-significant inverse correlation (*r* = −0.29) with initial levels or the level of acetyl-carnitine. 

### 3.11. Case Studies

There were also 3 exceptional cases of improvement during the study, all of which occurred in the treatment group.

#### 3.11.1. Case Study A

Increase in Physical Strength/Endurance/Energy: Participant A was a 9-year-old female with severe ASD, moderately overweight (BMI = 31.5), and very low strength, endurance, and energy level. She could not get in/out of the family van, climb stairs, or get up off the floor by herself, and had a low activity level overall. She could only walk a quarter mile before sitting and refusing to get up, so a wheelchair was used for outings. Around four months after treatment started her strength and endurance began to improve significantly, and by 6–12 months into the study she was able to get in/out of the van, walk up/down stairs, walk two miles, and attend outings without tiring. The wheelchair was put in storage and no longer needed. Her overall energy level increased substantially to the level when she was a toddler, and she began to skip around the house. Her diet had been self-limited with total avoidance of beef and pork products (the main dietary sources of carnitine), and her improvement seemed to primarily change with the addition of high-dose carnitine at 4 months into the study. An in-depth assessment of her carnitine status revealed that, averaging over measurements of 37 different types of carnitine (acetyl-carnitine species), her pre-treatment levels averaged only 68% of normal, and after treatment they averaged 18% above normal. So, low carnitine seems likely to have contributed to her challenges, and carnitine supplementation seems to have helped.

#### 3.11.2. Case Study B

Complete Resolution of Inability to Urinate: Participant B was a 27-year-old male with severe ASD and a history of severe urinary retention and occasional kidney stones for three years, requiring daily catheterization and occasional hospitalization. The cause was unknown and assumed to be neurological. Previous treatment with Flomax and Bethanecol was ineffective. The daily intermittent catheterization caused much discomfort to the subject, including bouts of urinary tract infections, bladder infections and irritation of the external urethral orifice, requiring numerous treatments with oral antibiotics and antifungals. His parents reported “His quality of life and social activity were much diminished. In addition, behavioral issues started to emerge, including an obsession with the constant touching of his genitals (probably initially caused by irritation which then evolved into a stimulative behavior). This became a huge problem when out in public, around peers and with his family members”.

As step one of a HGCSF diet, the subject was taken off all dairy products. Approximately four days after all dairy had been removed from his diet, the subject spontaneously went to the restroom and urinated on his own. He continued to be able to urinate on his own numerous times a day to the point where catheterization was no longer required. About three weeks after the subject had been taken off dairy products, he accidentally ate ice-cream, and the subject immediately ceased to be able to urinate on his own, returning to the necessity of daily intermittent catheterization. After approximately four days after eating the ice cream, the subject once again started spontaneously urinating on his own without assistance. He continued to be able to urinate on his own, eliminating the need for catheterization. Approximately four months after dairy had been removed from his diet, he accidentally ate cheese, upon which the subject once again lost the ability to urinate on his own, requiring intermittent catheterization. After approximately four days the subject started spontaneously urinating on his own again. The participant remained completely dairy-free for the remainder of the study, and continued to be able to urinate on his own and catheterization was no longer required, and there were zero episodes of kidney stones, urinary tract infections, bladder infections or urethral irritation. His parents reported that “his quality of life has improved dramatically and all behavior issues, including the constant touching of his genitals, have ceased. His social interactions with his peers and family members have improved dramatically and he is overall a much happier person”.

#### 3.11.3. Case Study C

Complete resolution of Pica: Participant C was a seven-year-old boy with severe pica. Within one week of starting the HGCSF diet there was a complete resolution of the pica which continued until the end of the study. Note that at baseline this boy had low levels of many nutrients compared to typical children, including: cobalamin (5% of normal), methylcobalamin (49%), beta-carotene (23%), nicotinic acid (29%), vitamin C (34%), pantothenic acid (35%), and riboflavin (52%). It seems likely that the severe pica was due to many significant nutritional deficiencies, and possibly a metabolic problem with cobalamin absorption or conversion.

## 4. Discussion

### 4.1. Blinded Evaluations (RIAS)

The significant improvement on the nonverbal IQ test suggests a substantial improvement in cognitive function. The verbal IQ did not change, perhaps because significant impairments in language remained. Since the RIAS evaluator was completely blinded, and the RIAS test is highly standardized, this increase in nonverbal IQ appears to be real. The improvement in nonverbal IQ is consistent with the much higher ratings of improvement in cognition on the PGI-2 for the treatment group vs. the non-treatment group (1.57 ± 0.9 vs. 0.38 ± 0.7). The magnitude of the effect is clinically significant: on the PGI-2 cognition question, 53% of treatment group were rated as better (39%) or much better (14%), vs. only 8% of the non-treatment group were rated as better, and none were rated much better.

### 4.2. Semi-Blinded Evaluations (VABS-II, CARS-2, SAS-Pro)

During the 12 months of treatment, the non-treatment group gained only 4 months of development on the VABS-II, consistent with a major developmental delay. In contrast, the treatment group gained an average of 18 months of development, with substantial improvements in many areas. However, their average developmental age still remained well below their biological age, so that they were still significantly impaired. This is consistent with modest but significant improvements on the CARS-2 and SAS-Pro.

### 4.3. Unblinded Parent/Self Evaluations (PDD-BI, ATEC, ABC, SRS, SSP, PGI-2, 6-GSI)

As mentioned previously, these unblinded evaluations are somewhat affected by a “placebo” effect, so the improvements reported represent an “upper bound” on the actual degree of benefit. The use of a non-treatment group provides some control for normal developmental improvement over the 12 months of the study. For the non-treatment group, it is interesting to note that there were some small improvements in several of the assessments (<10%, see Figure 2). However, the treatment group improved significantly more than the non-treatment group on all of the ASD/behavioral assessments. 

### 4.4. GI Symptoms

The treatment group had significantly more improvement than the non-treatment group on the 6-GSI, primarily due to improvements in constipation, diarrhea, and stool smell. This is consistent with the PGI-2 subscale for Stool/GI symptoms, which found that the treatment group improved much more than the non-treatment group (approximately “slightly better (0.94) vs. “no change (−0.11), see Table 11. This is also consistent with a significant reduction in constipation symptoms (−48% vs. −13%, *p* = 0.003) reported as one question on the ATEC (only evaluating those with initial mild, moderate, or severe constipation—19 in the treatment group, and 17 in the non-treatment group). The ATEC did not show a significant difference in diarrhea. Overall, these three assessments suggest that there was a significant improvement in GI symptoms in the treatment group compared to the non-treatment group.

### 4.5. Handgrip Strength

Both groups had only a small increase in handgrip strength, consistent with what might be expected by 12 months of development, but no significant difference between them and not enough to catch up to their peers.

### 4.6. Treatment Effectiveness and Continuation

The parents rated the vitamin/mineral supplement (started day 0) and the EFAs (started day 30) as the two most effective treatments, which is consistent with Figure 14 showing that most improvement occurred during the first three months of treatment. This is also consistent with those two treatments being rated by parents as the most likely treatments to continue. There was also a small increase in the PGI-2 between months 9–12, presumably due to the start of the healthy HGCSF diet on day 210. It should be noted that compliance with the healthy HGCSF diet was lower than for the other treatments, so benefit might have been somewhat larger if compliance was higher.

### 4.7. Medical Tests

The CBC, ChemPanel, and BMI results demonstrated that the treatment combination was generally safe, consistent with the modest number and modest intensity of adverse effects. There was no significant change in CRP, a general marker of inflammation, but levels at the start of the study were not significantly different between the ASD and neurotypical groups. 

#### 4.7.1. PUFAs

The PUFA test results demonstrate compliance with consumption of the EFA supplement, and demonstrate a large effect on PUFA levels in RBC. It should be noted that the EFA supplement included mostly omega-3 fatty acids (609 mg/capsule), but also some omega-6 fatty acids (198 mg/capsule). The percentage increase in EPA (+525%) was approximately 6 times higher than the increase in DHA (+83%), which is consistent with the composition of the fish oil used in this study (approximately 4:1 EPA:DHA). Conversely, there were small but significant decreases in linoleic acid, dihomo-γ-linolenic acid (DGLA), and arachidonic acid (AA); those small decreases may be partly due to competitive absorption with the omega-3 fatty acids, and partly due to competition for delta-5-saturase and delta-6-desaturases. Those desaturases are shared among omega-3, -6, and -9 fatty acids, and the desaturases have a preference in the order of omega 3-> omega-6 > omega-9. So, giving large amounts of omega-3 decreases the availability of the desaturases for omega-6 and omega-9, so less linoleic acid is converted to DGLA and AA. The decrease in arachidonic acid likely results in less production of pro-inflammatory eicosanoids, and that decrease is likely beneficial for some children with ASD. Similarly, the increase in oleic acid (non-significant) and significant decreases in elaidic and palmitoleic are also likely partly due to increased competition for the desaturases, so that less oleic is converted to elaidic and palmitoleic; elaidic and possibly palmitoleic are harmful trans-fats. It is also possible that the HGCSF diet, which recommended avoiding “junk food”, also helped decrease the level of elaidic acid, which is the most common trans-fat in hydrogenated vegetable oils.

Overall, the increases in EPA and DHA, and decreases in AA and elaidic acid, are very positive, and likely reduce inflammation throughout the body.

#### 4.7.2. Vitamins

The treatment group had significant improvements in the level of many vitamins (B2, B5, B6, folic acid, B12 (as cyanocobalamin), and CoQ10H_2_), but not all vitamins. This suggests that larger doses and/or more bioavailable forms of the other vitamins are needed to have a significant effect on blood levels, and larger doses/absorption may in some cases result in greater therapeutic benefit. It also suggests that the methylcobalamin was not absorbed, and that cyanocobalamin was not converted to methylcobalamin in plasma, but this conversion can occur intracellularly, and the improvement in homocysteine (which requires methyl-B12) suggests that this conversion occurred. For future studies we recommend increasing the level of vitamin D, since the current supplement did not increase levels significantly despite supplementation levels well above the Recommended Dietary Allowance (RDA) for vitamin D, and other studies of even higher doses of vitamin D for ASD reported significant benefits [75,76].

#### 4.7.3. Essential Elements (“Minerals”)

The treatment group had minor increases in selenium and chromium, and no other significant changes. There were no significant differences in the levels of essential elements between the ASD and neurotypical group at the start of the study, and only modest dosages of minerals were given in the vitamin/mineral supplement. Since the body can regulate levels of most essential minerals by adjusting absorption and excretion based on current mineral levels, it is not surprising that there was little significant effect of modest mineral supplementation when mineral levels on average are in the normal range.

#### 4.7.4. Homocysteine

Compared to the non-treatment group, the treatment group had a significant decrease in homocysteine, resulting in levels similar to (slightly below) the neurotypical group. This is presumably partly due to the supplementation with folinic acid and vitamin B12, which are enzymatic cofactors for recycling homocysteine to methionine, and partly due to supplementation with vitamin B6, which is the enzymatic co-factor for converting homocysteine to cystathionine, and for converting cystathionine to cysteine. 

#### 4.7.5. Carnitine

The carnitine test results demonstrated that both groups started at levels similar to the neurotypical controls, and the treatment resulted in a significant increase in l-carnitine, and a similar non-significant increase in acetyl-l-carnitine. Since the carnitine supplementation was primarily acetyl-l-carnitine (there was a small amount of l-carnitine in the vitamin/mineral supplement), this suggests inter-conversion between the two forms, as expected. However, the increase in plasma carnitine was only approximately 25%, which was less compared to another study [50] which used an equivalent dosage of l-carnitine and found a 70% increase in total carnitine in plasma, and another study using twice the dosage of l-carnitine found even higher increases in carnitine levels [51]. Comparing the results of the present study with the other two ASD studies suggests that l-carnitine may be better absorbed than acetyl-l-carnitine, and hence may be more effective. Indeed, l-carnitine supplementation was found to result in several significant improvements in symptoms in children with ASD in two other randomized, double-blind, placebo-controlled studies [50,51], whereas the benefit in this study seemed to be more modest (see Table 20). Also, initial levels of plasma carnitine were not good predictors of parents’ ratings of Treatment Effectiveness of carnitine—we hypothesize that intracellular levels may be better predictors.

#### 4.7.6. Digestive Enzymes

The digestive enzymes were not rated as highly as the three top-rated treatments. One randomized, double-blind study did find that digestive enzymes were helpful for autism [74], but another similar study did not find significant benefit [57]. So, it may be that the particular blend of digestive enzymes is important.

### 4.8. Case Studies

The three case studies suggest that nutritional deficiencies and/or food intolerances can have significant effects, and the comprehensive nutritional/dietary treatment protocol is a safe and effective way to identify and treat some intractable problems.

### 4.9. Age Effects

An evaluation of changes on all the outcome measures suggests that there was no significant correlation of benefits with age, so children and adults of all ages are likely to benefit from this combination treatment. 

### 4.10. Developmental History

An evaluation of changes on all the outcome measures for the early-onset group vs. the regression/plateau group did not reveal significant differences between the groups, but the sample size of each subgroup is small so only large differences could be observed. So, it appears that individuals with early-onset autism and regressive autism are both likely to benefit from this comprehensive nutritional treatment.

### 4.11. Age and Gender Sub-Analysis

For age, there was a small correlation of adjusted age vs. change in CARS (*r* = −0.43) and average PGI-R 2 (*r* = 0.40), suggesting the older participants improved somewhat more, but there was no correlation of adjusted age vs. the other measures (CARS, SRS, SSP, ATEC, ABC, PDD-BI, Vineland, PSAS, RIAS (VIX, NIX, CMX)). So, overall, age was not associated with degree of change, and all ages (children and adults) seemed to improve approximately the same. 

For gender, there were no significant differences on any of the behavioral assessments when comparing males vs. females. The males improved slightly more on ABC, RIAS (NIX), PDD-BI, and ATEC, and the females improved slightly more on SSP, PGI-R2, PSAS and Vineland, but these differences were not significant. The other behavioral assessments (ADOS, SRS, CARS, and RIAS (CMX, CIX and VIX)) were similar between males and females (less than 5% difference).

### 4.12. Limitations and Strengths

This study was designed to assess the possible benefits of a comprehensive nutritional and dietary intervention. A strength of the study is that it was a randomized, controlled study, but a major limitation of this study is that implementation of a healthy, HGCSF diet does not allow blinding of participants. The RIAS evaluation was single-blinded, and the CARS and SAS-Pro were semi-blinded (the evaluators were blinded, the participants were not), so those results are fairly robust. The parent evaluations certainly are subject to some placebo-effect but provide an upper-bound on possible benefits. The laboratory measurements were conducted in a blinded manner, so those results should be reliable.

A strength of this study is the long-term nature (12 months), allowing a fuller determination of possible benefits and adverse effects, which is important since the effect of nutritional interventions is likely slower than the effect of pharmaceutical interventions, and since individuals with autism often use nutritional supplements for long periods. The study was primarily designed to evaluate the cumulative effect of all of the treatments, but the sequential administration of treatments provided some insight into the relative merits of the individual treatments. A limitation of this study is its exploratory nature, so that sample size estimates were not made formally due to lack of information on the effect size of the combined treatment protocol.

A limitation of this study is that all participants received all treatments, whereas probably only a subset are likely to benefit from any single intervention (for example, only participants with low carnitine are likely to benefit from carnitine supplementation). Unfortunately, in many cases we do not have reliable biomarkers to accurately predict who is likely to benefit from a given intervention. So, this study used a comprehensive nutritional and dietary intervention approach, so that all participants were provided with all therapies, even though a given participant might only benefit from a subset of those interventions. Future studies could try to determine which treatments were most beneficial, using the results of this study to guide those future studies.

### 4.13. Relevance to Clinical Practice

The individual treatments used in this study have been used in previous studies, and were generally well-tolerated with minimal adverse effects. This study demonstrates that the combination of all of those treatments is feasible for most families, again with minimal adverse effects. The HGCSF diet had the lowest compliance, but still most families managed to implement the diet successfully. The number of pills required for all of the supplements was also a concern for some families, but splitting them up into 2–3 times/day made it manageable for most participants. Figure 15 demonstrates that most families wanted to continue with most treatments. So, we believe that the treatments used here should be considered for use in clinical practice for most children and adults with ASD.

### 4.14. Suggestions for Future Research

The results of this study strongly suggest that future similar studies are warranted, with a focus on vitamins/minerals, essential fatty acids, and HGCSF diet. A randomized double-blind placebo-controlled study design could be considered if the diet portion is not included. More extensive biochemical measurements, including metabolomics, may help determine how to optimize vitamin/mineral supplements and other treatments. For essential fatty acids, higher doses may be warranted, as even higher doses are used for improving cardiac health. For carnitine, switching to l-carnitine may improve benefit. For digestive enzymes, it appears more research is needed to improve them. For Epsom salt baths, it was previously shown that the vitamin/mineral supplement (with MSM, a source of sulfate) was only partially able to improve levels of plasma sulfate [15], and biochemical measures are needed to determine if the addition of the Epsom salt baths was sufficient to meet biological needs for sulfate, the fourth most abundant mineral in the body.

## 5. Conclusions

The study results suggest that the comprehensive nutrition/diet protocol was safe and effective. The nutritional supplements and healthy diet improved nutritional status, and hence presumably increased the brains ability to function and learn. This is supported by the increase in non-verbal IQ, and the substantial 18-month increase in developmental ability in communication, daily living skills, and social skills. Modest improvements in CARS-2 and SAS-Pro suggest some reduction in autism symptoms, consistent with parent reports of improvements on the PDD-BI, ATEC, and SRS. Parent reports also suggest improvements in aberrant behaviors (ABC—Irritability, Lethargy/Social Withdrawal, Stereotypy, and Hyperactivity), sensory processing (SSP), and GI symptoms (6-GSI, PGI-2, ATEC), and Overall (PGI-2). There was not a significant effect on handgrip strength. The treatment efficacy seemed to be similar for both genders and all ages, probably because nutritional requirements are similar for both genders and all ages (after normalizing for caloric intake).

The three unusual case reports, in which three very different long-term problems were greatly improved, shows the power of comprehensive nutritional interventions in addressing complex, puzzling medical conditions which may involve one or more nutritional deficiencies.

There were many significant increases in vitamins, essential fatty acids, and carnitine, and an improvement in homocysteine. The vitamin/mineral supplement and essential fatty acids appeared to have the most clinical benefit, although other treatments appeared to have some benefit for some individuals. So, this comprehensive treatment approach is recommended as a promising therapy for children and adults with ASD, with an emphasis on the vitamin/mineral supplement and essential fatty acids as probably being the most helpful.

The data also suggests some possible improvements could be made to the treatment combination. Specifically, it appears that l-carnitine may be better absorbed than acetyl-l-carnitine. Also, although many vitamins were well-absorbed, larger doses and/or more bioavailable forms of the other vitamins are needed to have a significant effect on blood levels, and larger doses/absorption may in some cases result in greater therapeutic benefit. So, it seems likely that the current treatment protocol could be further improved by making these changes.

## Figures and Tables

**Table 1:** Participants.

	ASD–Treatment	ASD–Non–Treatment	Neurotypical
Total Participants	37	30	50
Male	30 (81%)	25 (83%)	41 (82%)
Female	7 (19%)	5 (17%)	9 (18%)
Age (years)	10.8 ± 7.0	12.3 ± 10.1	12.2 ± 7.5
Children (ages 3–12)	Children *n* = 28 (76%)	Children *n* = 20 (67%)	Children *n* = 34 (68%)
Teens (ages 13–20)	Teens *n* = 6 (16%)	Teens *n* = 7 (23%)	Teens *n* = 11 (22%)
Adults (ages 20+)	Adults *n* = 3 (8%)	Adults *n* = 3 (10%)	Adults *n* = 5 (10%)
Diagnosis	Autism = 29 (83%)	Autism = 21 (70%)	
	Asperger’s = 3 (9%)	Asperger’s = 5 (17%)	
	PDD-NOS = 3 (9%)	PDD-NOS = 4 (13%)	
Autism Onset	Regressive = 13 (36%)	Regressive = 9 (31%)	
	Plateau = 8 (22%)	Plateau = 10 (34%)	
	Early Onset = 15 (42%)	Early Onset = 10 (34%)	
Asthma	9 (25%)	8 (27%)	8 (16%)
Food Allergies	13 (36%)	3 (10%)	2 (4%)
Other Allergies	19 (51%)	13 (43%)	15 (30%)
Other Health Issues—frequency	15 (41%)	14 (47%)	2 (4%)
Other Health Issues—description (note: these are likely under-reported since we only asked a general question about “other health conditions”, and some of these symptoms might be viewed as part of autism)	ADHD-4; sensory problems-3; intellectual disability-2; seizures; early puberty; vascular malformation; mood disorder; spinal fusion; agenesis of lung; gastritis; eczema; apraxia; type 2 diabetes	ADHD-6, cerebral palsy-3, hypotonia-2, learning disability-2, depression-2, dysphagia, sensory disorder, reflux, seizures, sleep disorder, sexual prematurity, type 1 diabetes, OCD, anxiety	nocturnal enuresis; Hashimoto’s thyroiditis
Medications (participants taking one or more of the different types of medications)	allergy-6; psych-4; asthma-3; seizure-2; sleep-2; diabetes-1; cholesterol-1; laxative-1	psych-8; allergy-4; seizure-2; thyroid-2; sleep-2; blood pressure-1; diabetes-1; acne-1	Allergies-2; asthma-1; thyroid-1

**Table 2:** Vitamin/Mineral Supplement.

Ingredients	Amount
Vitamin A (85% beta carotene and 15% palmitate, IU)	6500
Vitamin C (from calcium ascorbate, mg)	500
Vitamin D3 (cholecalciferol, IU)	1000
Vitamin E (as alpha-tocopherol, IU)	150
Vitamin K (K1 and K2, mcg)	55
Vitamin B1 (thiamin hydrochloride, mg)	20
Vitamin B2 Riboflavin (mg)	40
Niacin (71% inositol hexanicotinate & 29% niacinamide, mg)	35
Vitamin B6 (50% as P5P pyridoxal 5 phosphate, 50% as pyridoxine hydrochloride, mg)	40
Folate (as folic acid, folinic acid, and l-5-methyltetrahydrofolate, mcg)	600
Vitamin B12 (50% as methylcobalamin & 50% as cyanocobalamin, mcg)	500
Biotin (mcg)	225
Pantothenic Acid (calcium d-pantothenate, mg)	30
Iodine (potassium iodide, mcg)	100
Lithium (mcg)	350
Choline (from choline bitartrate, mg)	250
Inositol (mg)	100
Calcium (mg)	70
Magnesium (magnesium citrate, mg)	100
Zinc (zinc gluconate, mg)	15
Selenium (selenomethionine and sodium selenite, mcg)	40
Manganese (manganese amino acid chelate, mg)	1
Chromium (chromium amino acid chelate, mcg)	70
Molybdenum (sodium molybdate dihydrate, mcg)	100
Potassium (from potassium chloride, mg)	50
MSM (methylsulfonylmethane, mg)	500
Vitamin E as mixed tocopherols (mg)	100
CoQ10 (mg)	50
*N*-acetyl-cysteine (mg)	45
Acetyl-l-carnitine (mg)	200
Vanadium (mcg)	25
Boron (mcg)	250

This is the dosage for a 60-pound (27 kg) child. Dosage was adjusted up/down by bodyweight.

**Table 3:** Digestive Enzyme Ingredients (1 capsule).

Ingredients	
Amylase	3500 DU
Peptidase	13,000 HUT
Glucoamylase	50 AGU
Xylanase	7000 XU
Protease 4.5	22,000 HUT
Protease 3.0	35 SAPU
Amylase	1500 DU
Invertase	800 SU
Alpha-galactosidase	100 GalU
Lactase	500 ALU
Lipase	500 FIP

**Table 4:** Professional Evaluations.

	Treatment Group (*n* = 28)					Non-Treatment Group (*n* = 27)					*t*-Test
	Initial	S.D.	Final	S.D.	% Change	Initial	S.D.	Final	S.D.	% Change	
RIAS											
Nonverbal Intelligence Index	69.6	23	76.3	24	+10%	85.8	24	85.3	24	−1%	0.01
Verbal Intelligence Index	63.1	24	66.0	26	+5%	77.6	24	81.5	25	+5%	n.s.
Composite Memory Index	71.3	20	75.3	23	+6%	81.0	23	87.9	25	+8%	n.s.
CARS-2	39.3	5.6	33.9	7.0	−22% #	38.2	5.2	35.0	5.8	−14% #	0.03
SAS-Pro	6.9	1.7	6.0	2.1	−13%	6.0	1.9	5.6	2.3	−6%	0.04

*t*-test values were considered not significant (n.s.) if they were above a *p*-value of 0.1. #- the percent change in CARS-2 calculated based on minimum possible score of 15.

**Table 5:** Vineland Adaptive Behavior Scales II (VABS-II).

	Treatment Group (*n* = 19)					Non-Treatment Group (*n* = 16)					*t*-Test
	Initial	S.D.	Final	S.D.	% Change	Initial	S.D.	Final	S.D.	% Change	
Communication	4.5	1.8	5.7	2.5	+27%	5.5	2.9	5.8	2.7	+5%	0.01
Receptive	3.3	2.3	4.8	2.8	+43%	3.8	2.1	4.5	2.6	+17%	0.09
Expressive	3.6	1.7	4.8	2.5	+34%	4.5	2.8	4.9	2.9	+9%	0.06
Written	6.6	2.3	7.6	2.7	+16%	8.2	4.1	8.1	3.1	−2%	0.03
Daily Living Skills	5.3	3.4	6.9	3.7	+31%	7.4	5.0	7.7	4.6	+4%	0.007
Personal	4.9	3.6	6.5	4.3	+34%	7.5	5.5	8.3	5.6	+11%	n.s.
Domestic	4.8	3.6	6.9	3.7	+44%	7.0	4.5	7.0	4.3	−1%	0.002
Community	6.1	4.1	7.1	3.7	+18%	8.2	5.5	8.4	4.5	+2%	0.07
Social	4.4	2.8	6.2	3.8	+39%	5.5	3.9	5.9	3.4	+8%	0.05
Interpersonal Relationships	3.5	2.5	5.6	4.2	+59%	3.9	3.1	3.9	2.6	+2%	0.01
Play and Leisure Time	4.3	2.6	5.4	2.7	+25%	5.4	3.5	6.9	4.2	+28%	n.s.
Coping Skills	5.5	3.9	7.5	5.3	+37%	7.1	5.8	6.8	4.8	−5%	0.03
Motor Skills	4.4	1.3	5.1	1.0	+15%	5.1	1.6	5.7	1.0	+12%	n.s.
Gross Motor	4.0	1.3	4.5	1.2	+13%	4.8	1.6	5.6	1.3	+16%	n.s.
Fine Motor	4.8	1.6	5.6	1.2	+17%	5.4	1.6	5.8	1.0	+9%	n.s.
Average VABS (excluding motor skills)	4.7	2.5	6.3	3.2	+32%	6.1	3.7	6.5	3.4	+6%	0.008

Units are developmental age in years. *t*-test values were considered not significant (n.s.) if they were above a *p*-value of 0.1.

**Table 6:** Pervasive Developmental Disorders Behavior Inventory (PDD-BI).

	Treatment Group (*n* = 27)					Non-Treatment Group (*n* = 26)					*t*-Test
	Initial	S.D.	Final	S.D.	% Change	Initial	S.D.	Final	S.D.	% Change	
Maladaptive Behaviors—higher scores mean worse problems											
Sensory	24.4	14.1	18.7	12.3	−23%	14.4	11.0	13.7	11.2	−5%	0.001
Ritual	18.9	7.2	13.9	7.6	−27%	15.4	7.8	15.1	8.1	−2%	0.001
SOCPP	20.6	7.8	16.1	8.2	−22%	17.3	6.7	16.3	6.6	−6%	0.008
SEMPP	20.1	7.8	17.3	7.7	−14%	15.2	6.8	14.5	6.5	−5%	0.08
AROUSE	20.7	7.2	16.3	7.8	−21%	17.4	7.2	17.0	7.2	−2%	0.002
FEARS	23.4	9.0	18.7	9.4	−20%	24.4	8.1	23.4	8.8	−4%	0.03
AGG	18.6	12.4	14.2	11.0	−24%	14.0	11.0	14.8	11.2	5%	0.002
Adaptive Behaviors—higher scores mean higher ability											
SOCAPP	62.8	22.2	74.2	23.3	+18%	68.2	20.1	73.3	17.3	+8%	0.02
EXPRESS	57.4	24.7	66.1	24.2	+15%	66.8	24.3	70.5	21.0	+5%	0.004
LMRL	27.4	8.9	29.6	8.6	+8%	27.1	8.6	28.1	7.9	+4%	0.07
Modified Autism Composite	−56.3	57.7	−91.6	60.7	−21% #	−85.2	53.0	−96.0	50.2	−5% #	0.0002

# Since the Autism Composite is not rated on an absolute scale, we report the average of the % changes of each of the subscales of which it is composed, to give a sense of the degree of change of symptoms.

**Table 7:** Autism Treatment Evaluation Checklist (ATEC).

	Treatment Group (*n* = 27)					Non-Treatment Group (*n* = 26)					*t*-Test
	Initial	S.D.	Final	S.D.	% Change	Initial	S.D.	Final	S.D.	% Change	
Speech Communication	9.4	5.8	6.0	4.8	−36%	7.0	6.5	6.0	5.9	−14%	0.0007
Sociability	16.5	8.2	11.4	7.1	−31%	15.9	5.9	15.2	7.0	−5%	0.003
Sensory/Cognitive Awareness	16.6	6.6	11.7	5.6	−30%	12.3	6.9	11.6	6.8	−6%	0.00002
Health/Physical/Behavior	28.4	11.7	21.7	12.0	−24%	22.9	7.6	22.1	7.6	−4%	0.0009
Total ATEC Score	70.9	25.7	50.8	24.2	−28%	58.2	20.7	54.8	22.7	−6%	0.00004

Higher scores mean worse problems.

**Table 8:** Aberrant Behavior Checklist.

	Treatment Group (*n* = 27)					Non-Treatment Group (*n* = 25)					*t*-Test
	Initial	S.D.	Final	S.D.	% Change	Initial	S.D.	Final	S.D.	% Change	
Irritability	16.0	10.0	12.8	9.2	−20%	12.0	10	11.6	10.0	−4%	0.02
Lethargy/Social Withdrawal	14.9	10.6	10.1	8.5	−32%	14.1	7.6	12.5	7.3	−11%	0.01
Stereotypy	7.6	4.9	5.2	4.1	−31%	6.5	4.8	5.7	4.9	−12%	0.01
Hyperactivity	24.2	13.0	18.3	11.7	−24%	18.5	13	18.0	12.9	−3%	0.0001
Inappropriate Speech	6.4	3.3	5.1	3.1	−21%	4.0	2.9	3.6	2.9	−9%	0.05
Total ABC Score	68.9	33.5	51.4	31.7	−26%	55.0	27	51.4	27.5	−7%	0.001

Higher scores mean worse problems.

**Table 9:** Social Responsiveness Scale (SRS).

	Treatment Group (*n* = 27)					Non-Treatment Group (*n* = 25)					*t*-Test
	Initial	S.D.	Final	S.D.	% Change	Initial	S.D.	Final	S.D.	% Change	
Awareness	15.0	4.1	13.4	4.0	−11%	12.4	2.7	11.7	2.5	−6%	n.s.
Cognition	21.4	6.4	18.6	7.4	−13%	18.8	4.1	18.3	4.5	−3%	0.01
Communication	38.8	11.6	33.2	12.4	−14%	34.5	6.8	33.3	8.0	−4%	0.01
Motivation	17.3	6.1	14.5	6.1	−16%	17.6	4.7	16.8	4.7	−5%	0.03
Mannerisms	23.3	6.1	20.0	7.0	−14%	20.7	6.4	20.6	6.8	0%	0.02
Total SRS	115.7	30.6	99.7	33.7	−14%	104.0	18.2	100.6	21.4	−3%	0.01

Higher scores mean worse problems. *t*-test values were considered not significant (n.s.) if they were above a *p*-value of 0.1.

**Table 10:** Short Sensory Profile.

	Treatment Group (*n* = 27)					Non-Treatment Group (*n* = 26)					*t*-Test
	Initial	S.D.	Final	S.D.	% Change	Initial	S.D.	Final	S.D.	% Change	
Tactile Sensitivity	22.7	5.9	26.4	5.2	+16%	24.1	4.9	25.2	5.1	+4%	0.007
Taste/Smell Sensitivity	10.5	6.0	11.8	5.3	+12%	11.1	5.4	11.5	5.5	+3%	0.05
Movement Sensitivity	11.2	4.4	11.7	4.0	+4%	10.8	3.5	10.6	3.3	−1%	0.06
Underresponsiveness/Seeks Sensation	17.9	6.9	20.5	5.5	+14%	22.0	7.1	22.3	7.3	+2%	0.006
Auditory Filtering	16.2	4.7	18.9	5.1	+16%	16.2	5.5	16.0	5.3	−1%	0.0003
Low Energy/Weak	19.3	7.5	20.4	7.3	+6%	17.5	7.1	17.9	7.4	+2%	n.s.
Visual/Auditory Sensitivity	14.9	4.8	16.9	4.5	+13%	16.0	4.1	16.0	4.2	0%	0.004
Total SSP	113	27	127	27	+12%	118	20	120	21	+2%	0.0003

Higher scores mean less sensory problems. *t*-test values were considered not significant (n.s.) if they were above a *p*-value of 0.1.

**Table 11:** Parent Global Impressions 2 (PGI 2).

		Treatment *n* = 28			Non-Treatment *n* = 26		*t*-Test
	*n* for Each Symptom at Start of Study	Change	S.D.	*n* for Each Symptom at Start of Study	Change	S.D.	
Expressive Language/Speech	27	1.7	0.8	25	0.3	0.9	0.0000006
Receptive Language/Comprehension	26	1.9	0.9	26	0.3	0.7	0.000000003
Play Skills	27	1.5	0.8	24	0.3	0.8	0.0000008
Cognition Thinking	28	1.6	0.9	24	0.4	0.7	0.000003
Attention Focus	28	1.5	1.1	25	0.3	0.9	0.00006
Stools/GI Issues	24	0.9	1.5	19	−0.1	0.6	0.003
Sleep	22	0.9	1.2	16	−0.2	1.2	0.01
Sociability	28	1.4	1.0	25	0.1	0.8	0.000009
Hyperactivity	23	0.9	1.0	16	−0.3	0.6	0.00008
Tantruming	23	1.2	1.3	19	−0.1	1.3	0.003
Eye Contact	27	1.4	0.9	25	0.2	0.8	0.000003
Mood/Happines	24	1.6	1.0	23	0.1	0.9	0.000003
Anxiety	27	0.9	1.3	25	−0.2	1.0	0.002
Stimming/Preservation	28	1.1	1.0	22	−0.1	0.8	0.00001
Sound Sensitivity	26	1.0	1.0	23	0.1	0.5	0.0002
Aggression	21	0.9	1.2	16	−0.3	1.3	0.01
Self-Abusive	16	0.8	1.4	12	−0.2	1.5	0.089
Overall	28	1.68	0.77	26	0.27	0.92	0.0000002
Average	28	1.2	0.7	27	0.1	0.5	0.00000003

**Table 12:** 6-Item Gastrointestinal Symptom Index (6-GSI).

	Treatment Group (*n* = 22)					Non-Treatment Group (*n* = 20)					*t*-Test
	Initial	S.D.	Final	S.D.	% Change	Initial	S.D.	Final	S.D.	% Change	
Constipation	0.6	0.8	0.4	0.5	−43%	1.1	0.8	1.0	0.7	−14%	n.s.
Diarrhea	0.3	0.6	0.1	0.3	−67%	0.1	0.2	0.0	0.0	−100%	n.s.
Average Stool Consistency	0.4	0.5	0.3	0.5	−13%	0.3	0.5	0.2	0.4	−50%	n.s.
Stool Smell	0.7	0.8	0.4	0.6	−44%	0.8	0.9	0.9	1.0	+20%	0.020
Flatulence	1.0	0.8	0.9	0.8	−13%	1.1	0.8	1.0	0.9	−10%	n.s.
Abdominal Pain	0.3	0.6	0.3	0.6	−14%	0.4	0.5	0.4	0.6	−13%	n.s.
Total Severity Score	3.4	1.8	2.4	1.8	−30%	3.7	1.8	3.3	2.2	−10%	0.050

Since only a subset of participants had GI problems, we only included them in this analysis if they had non-zero Total Severity Scores at the start of the study. *t*-test values were considered not significant (n.s.) if they were above a *p*-value of 0.1.

**Table 13:** Handgrip Strength.

	Treatment Group (*n* = 28)					Non-Treatment Group (*n* = 25)					*t*-Test
	Initial	S.D.	Final	S.D.	% Change	Initial	S.D.	Final	S.D.	% Change	
Handgrip Strength	40.3	26.6	41.8	21.5	+4%	44.8	24.9	46.7	22.5	+4%	n.s.

*t*-test values were considered not significant (n.s.) if they were above a *p*-value of 0.1.

**Table 14:** Complete Blood Count.

	Treatment Group (*n* = 25)					Non-Treatment Group (*n* = 21)					*t*-Test
	Initial	S.D.	Final	S.D.	% Change	Initial	S.D.	Final	S.D.	% Change	
WBC (×10^3^/uL)	6.86	2.02	6.88	2.41	0%	6.08	1.57	5.79	1.57	−5%	n.s.
RBC (×10^6^/uL)	4.98	0.50	4.89	0.52	−2%	4.92	0.31	4.93	0.28	0%	0.04
Hemogoblin (g/dL)	14.2	1.44	14.1	1.55	−1%	14.1	1.12	14.2	1.10	+1%	n.s.
Hematocrit (%)	41.4	3.91	41.2	4.26	−1%	41.3	2.92	41.4	2.73	0%	n.s.
MCV (fL)	83.4	5.70	84.4	5.96	+1%	84.0	2.78	84.0	2.94	0%	0.02
MCH (pg)	28.5	2.09	28.8	2.23	+1%	28.6	1.33	28.7	1.33	0%	n.s.
MCHC (g/dL)	34.2	0.71	34.1	0.76	−0%	34.1	0.81	34.2	0.96	0%	n.s.
RDW (%)	13.7	0.68	13.5	0.71	−1%	13.6	0.77	13.5	0.69	−1%	n.s.
Platelets (×10^3^/uL)	319	75.5	311	87.3	−3%	285	59.7	296	72.9	4%	n.s.
Neutrophils (Absolute) (×10^3^/uL)	3.34	1.46	3.58	1.88	+7%	2.86	1.05	2.70	0.93	−6%	n.s.
Lymphs (Absolute) (×10^3^/uL)	2.65	0.94	2.47	1.03	−7%	2.46	0.93	2.40	0.85	−3%	n.s.
Monocytes (Absolute) (×10^3^/uL)	0.56	0.16	0.58	0.19	+4%	0.50	0.16	0.45	0.14	−11%	0.08
Eos (Absolute) (×10^3^/uL)	0.27	0.30	0.21	0.21	−24%	0.24	0.17	0.21	0.16	−12%	n.s.
Baso (Absolute) (×10^3^/uL)	0.04	0.05	0.02	0.04	−33%	0.01	0.04	0.01	0.04	0%	n.s.
Immature Granulocytes (%)	0.00	0.00	0.00	0.00		0.00	0.00	0.00	0.00		
Immature Grans (Absolute) (×10^3^/uL)	0.00	0.00	0.00	0.00		0.00	0.00	0.00	0.00		

*t*-test values were considered not significant (n.s.) if they were above a *p*-value of 0.1.

**Table 15:** Blood Chemistry.

	Treatment Group (*n* = 25)					Non-Treatment Group (*n* = 21)					*t*-Test
	Initial	S.D.	Final	S.D.	% Change	Initial	S.D.	Final	S.D.	% Change	
Glucose, Serum (mg/dL)	87.6	5.63	90.0	8.98	+3%	92.7	28.1	95.6	21.9	+3%	n.s.
BUN (mg/dL)	13.5	3.77	11.3	3.77	−16%	13.3	3.29	14.0	3.56	+5%	0.01
Creatinine, Serum (mg/dL)	0.61	0.22	0.62	0.21	0%	0.61	0.18	0.65	0.21	+6%	n.s.
BUN/Creatinine Ratio	25.0	11.8	20.3	8.53	−19%	23.4	9.83	24.0	12.1	+3%	0.04
Sodium, Serum (mmol/L)	139	2.39	140	2.64	+1%	139	4.04	139	2.19	0%	n.s.
Potassium, Serum (mmol/L)	4.28	0.28	4.01	0.25	−6%	4.09	0.32	4.05	0.30	−1%	0.02
Chloride, Serum (mmol/L)	101	2.60	101	2.64	0%	102	2.75	103	2.68	+1%	n.s.
Carbon Dioxide, Serum (mmol/L)	19.7	2.04	20.7	2.36	+5%	20.5	1.82	20.4	2.72	−0%	n.s.
Calcium, Serum (mmol/L)	10.1	0.43	9.96	0.41	−2%	10.1	0.49	9.8	0.62	−4%	n.s.
Protein, Total, Serum (g/dL)	7.07	0.47	7.22	0.49	+2%	7.04	0.47	7.08	0.51	+1%	n.s.
Albunim spp., Serum (g/dL)	4.49	0.40	4.58	0.31	+2%	4.33	0.38	4.54	0.21	+5%	n.s.
Globulin, Total (g/dL)	2.58	0.36	2.64	0.37	+2%	2.71	0.58	2.55	0.41	−6%	0.02
A/G Ratio	1.78	0.33	1.77	0.27	−1%	1.70	0.47	1.83	0.28	+7%	n.s.
Bilirubin, Total (mg/dL)	0.36	0.23	0.46	0.35	+29%	0.34	0.12	0.34	0.12	0%	0.08
Alkaline Phosphatase, S (IU/L)	204	90.0	184	87.1	−10%	221	111	200	92.2	−9%	n.s.
AST (SGOT) (IU/L)	26.2	6.74	28.4	12.8	+8%	24.7	6.38	23.4	7.43	−6%	n.s.
ALT (SGPT) (IU/L)	22.1	10.5	26.1	21.2	+18%	18.9	9.58	20.5	11.9	+9%	n.s.
Ammonia, Plasma (ug/dL)	79.3	39.57	76.70	26.06	−3%	64.1	29.5	76.0	23.4	+19%	n.s.
Creatine Kinase, Total, Serum (U/L)	117	49.7	111	55.0	−6%	102	38.3	113	56.3	+11%	n.s.
Lactic Acid, Plasma (mg/dL)	16.9	10.1	16.7	12.5	−1%	13.0	6.22	13.3	11.4	+2%	n.s.
TSH (uIU/mL)	2.30	0.99	2.55	1.30	+11%	2.11	1.07	2.16	0.94	+2%	n.s.
Triiodothyronine, Free, Serum (pg/mL)	4.07	0.50	4.12	0.66	+1%	3.95	0.58	4.05	0.63	+3%	n.s.
T4, Free (Direct) (ng/dL)	1.33	0.15	1.32	0.14	−1%	1.27	0.19	1.21	0.20	−5%	n.s.

*t*-test values were considered not significant (n.s.) if they were above a *p*-value of 0.1.

**Table 16:** Red Blood Cell Fatty Acids.

	Average—Treatment (*n* = 25)					Average—Non-Treatment (*n* = 24)					*t*-Test
	Initial	SD	Final	SD	% Change	Initial	SD	Final	SD	% Change	
Arachidonic Acid (μmol/L)	910	89	727	99	−20%	911	66	899	99	−1%	0.0000001
Dihomo-g-linolenic (μmol/L)	95	22	84	16	−12%	95	22	97	17	+2%	0.003
Docosahexaenoic acid (μmol/L)	175	58	320	78	+83%	183	54	207	54	+13%	0.000000001
Eicosapentaenoic (μmol/L)	21	13	130	60	+525%	17	7.4	20	8.0	+22%	0.000000001
Elaidic (μmol/L)	8.7	1.8	6.4	1.7	−26%	8.3	2.3	7.3	1.7	−12%	0.03
Linoleic (μmol/L)	670	93	568	90	−15%	622	98	617	77	−1%	0.0001
Oleic (μmol/L)	650	77	725	75	+11%	619	35	670	44	+8%	n.s.
Palmitelaidic (μmol/L)	0.92	0.23	0.76	0.17	−17%	0.89	0.23	0.74	0.20	−17%	n.s.
Palmitic (μmol/L)	1194	108	1197	75	0%	1136	101	1153	65	+1%	n.s.
Palmitoleic (μmol/L)	10.9	3.9	9.6	4.4	−13%	11.2	4.2	12.4	5.4	+11%	0.01
Stearic (μmol/L)	885	82	833	40	−6%	872	55	862	55	−1%	0.08

*t*-test values were considered not significant (n.s.) if they were above a *p*-value of 0.1.

**Table 17:** Vitamin Levels.

	Average—Treatment (*n* = 27)					Average—Non-Treatment (*n* = 26)					*t*-Test
	Initial	S.D.	Final	S.D.	% Change	Initial	S.D.	Final	S.D.	% Change	
Vit B2 (riboflavin)	0.69	0.22	2.53	1.36	+268%	1.18	0.68	0.98	0.67	−17%	0.00000001
Vit B3 (niacinamide)	1.31	0.85	1.34	0.87	+2%	0.99	0.43	1.14	0.47	+14%	n.s.
Vit B5 (pantothenic acid)	1.01	1.28	4.54	3.21	+351%	0.86	0.65	1.63	1.34	+90%	0.0002
Vit B6 (4-pyridoxic acid)	1.21	1.30	6.45	3.85	+435%	1.21	1.29	1.29	1.07	+6%	0.000001
Vit B6 (pyridoxine)	1.21	0.55	1.51	0.73	+25%	1.26	0.75	0.85	0.42	−33%	0.008
Folic acid	0.65	0.61	1.42	1.07	+119%	1.10	1.25	0.73	1.86	−34%	0.02
B12-(cyanocobalamin)	0.86	0.49	1.24	0.54	+44%	0.94	0.41	0.90	0.26	−5%	0.006
B12-(methylcobalamin)	0.98	0.49	1.08	0.57	+9%	0.96	0.34	0.99	0.44	+3%	n.s.
Vit C (ascorbic acid)	0.79	0.32	1.18	0.56	+48%	0.81	0.49	1.01	0.50	+24%	n.s.
Vit D3	1.03	0.63	1.12	0.44	+9%	0.85	0.29	1.00	0.37	+18%	n.s.
Vit K2	0.97	0.55	1.22	0.47	+26%	0.83	0.54	1.09	0.44	+32%	n.s.
Choline	1.08	0.41	0.99	0.30	−8%	1.01	0.27	0.88	0.24	−12%	n.s.
Myoinositol	1.25	0.46	1.48	0.74	+19%	0.98	0.27	1.05	0.40	+7%	n.s.
CoQ10 (oxidized)	1.35	0.86	1.18	0.64	−13%	1.30	0.65	1.49	0.97	+15%	n.s.
CoQ10 (reduced form, CoQ10H2)	0.94	0.62	1.63	0.79	+72%	1.31	0.85	1.05	0.66	−20%	0.001

Vitamin levels as measured by a semi-quantitative metabolomics method, so results are normalized to levels in un-supplemented neurotypical controls (i.e., a level of 1.0 is the average of the neurotypical controls). *t*-test values were considered not significant (n.s.) if they were above a *p*-value of 0.1.

**Table 18:** RBC Elements.

	Treatment Group (*n* = 26)					Non-Treatment Group (*n* = 24)					*t*-Test
	Initial	S.D.	Final	S.D.	% Change	Initial	S.D.	Final	S.D.	% Change	
Ca (μg/g)	18.0	4.0	17.0	3.0	−5%	17.0	4.0	17.0	5.0	+3%	n.s.
Mg (μg/g)	46.0	4.0	46.0	5.0	0%	46.0	4.0	48.0	4.0	+3%	n.s.
K (mEq/L)	78.0	4.0	78.0	4.0	0%	78.0	5.0	77.0	3.0	−1%	n.s.
P (μg/g)	599	36.0	580	43.0	−3%	597	50.0	581	44.0	−3%	n.s.
Cu (μg/g)	0.7	0.1	0.7	0.1	+7%	0.7	0.1	0.7	0.1	+4%	n.s.
Zn (μg/g)	9.6	1.5	9.6	1.5	0%	9.5	1.1	9.5	1.2	−1%	n.s.
Fe (μg/g)	865	47.0	858	30.0	−1%	865	47.0	853	37.0	−1%	n.s.
Mn (μg/g)	0.02	0.01	0.02	0.01	+2%	0.02	0.01	0.02	0.01	+5%	n.s.
Cr (μg/g)	0.0005	0.0003	0.0006	0.0003	+12%	0.0005	0.0003	0.0004	0.0001	−15%	0.05
Se (μg/g)	0.3	0.03	0.3	0.04	+5%	0.3	0.04	0.2	0.03	−8%	0.001

*t*-test values were considered not significant (n.s.) if they were above a *p*-value of 0.1.

**Table 19:** Homocysteine Pathway.

	Treatment Group (*n* = 26)					Non-Treatment Group (*n* = 22)					*t*-Test
	Initial	S.D.	Final	S.D.	% Change	Initial	S.D.	Final	S.D.	% Change	
Cysteine (μM/dL)	24.5	4.1	24.1	3.8	−2%	24.4	2.7	24.2	3.1	−1%	n.s.
Homocysteine (μM/L)	7.1	2.7	5.0	1.4	−29%	6.1	1.7	5.7	1.4	−7%	0.00002
Methionine (μM/dL)	2.4	0.5	2.4	0.8	0%	2.2	0.5	2.6	0.6	+17%	0.09

*t*-test values were considered not significant (n.s.) if they were above a *p*-value of 0.1.

**Table 20:** Carnitine levels in plasma.

	Treatment Group (*n* = 27)					Non-Treatment Group (*n* = 26)					*t*-Test
	Initial	S.D.	Final	S.D.	% Change	Initial	S.D.	Final	S.D.	% Change	
l-carnitine	1.10	0.32	1.32	0.41	+20%	1.12	0.31	1.07	0.29	−5%	0.03
Acetyl-l-carnitine	1.05	0.42	1.39	0.47	+32%	0.96	0.53	1.01	0.39	+4%	n.s.

Units are normalized to those of neurotypical controls of similar age and gender (i.e., neurotypical group has levels of 1.0). For the treatment group, we exclude the 1 participant who did not tolerate the carnitine supplement. *t*-test values were considered not significant (n.s.) if they were above a *p*-value of 0.1.

## Abbreviations

6-GSI	6-item Gastrointestinal Severity Index
AA	Arachidonic Acid
ABC	Aberrant Behavior Checklist
ADHD	Attention Deficit Hyperactivity Disorder
ADOS	Autism Diagnostic Observation Schedule
AGG	Aggressiveness
AROUSE	Arousal Regulation Problems
ASD	Autism Spectrum Disorder
ASU	Arizona State University
ATEC	Autism Treatment Evaluation Checklist
ATP	Adenosine Triphosphate
BMI	Body Mass Index
BUN	Blood Urea Nitrogen
CARS-2	Childhood Autism Rating Scale 2
CBC	Complete Blood Count
CGI	Clinical Global Impressions
CLIA	Clinical Laboratory Improvements Amendments
CRP	C-Reactive Protein
DGLA	Dihomo-Gamma-Linolenic Acid
DHA	Docosahexaenoic Acid
EFA	Essential Fatty Acids
EPA	Eicosapentaenoic Acid
GABA	Gamma-Aminobutyric Acid
GFCF	Gluten-Free, Casein-Free
GLA	Gamma-Linolenic Acid
HGCSF	Healthy Gluten-Free Casein-Free Soy Free
IRB	Institutional Review Board
LC-MS/MS	Liquid Chromatography Tandem Mass Spectrometry
LMRL	Learning, Memory, and Receptive Language
MCV	Mean Corpuscular Volume
n.s.	Not Significant
NADH	Nicotinamide Adenine Dinucleotide
NADPH	Nicotinamide Adenine Dinucleotide Phosphate
PDD-BI	Pervasive Developmental Disorders Behavior Inventory
PDD-NOS	Pervasive Developmental Disorder-Not Otherwise Specified
PGI-2	Parent Global Impressions-2
PUFA	Polyunsaturdated Fatty Acid
RBC	Red Blood Count
RIAS	Reynolds Intellectual Assessment Scales
S.D.	Standard Deviation
SAM	*S*-Adenosylmethionine
SAS-Pro	Severity of Autism Scale
SEMPP	Semantic/Pragmatic Problems
SOCPP	Social Pragmatic Problems
SRS	Social Responsiveness Scale
SSP	Short Sensory Profile
VABS-II	Vineland Adaptive Behavior Scale II
